# Explanatory limitations of CSF, plasma, and MRI biomarkers for inflammation and blood–brain barrier disruption in dementia

**DOI:** 10.3389/fragi.2026.1923046

**Published:** 2026-09-11

**Authors:** Azadeh Golduzian, Erik B. Erhardt, Arvind Caprihan, John C. Adair, Janice E. Knoefel, Jill Prestopnik, Sasha Hobson, Sephira Ryman, Andrei Vakhtin, Kiran Bhaskar, Gary A. Rosenberg

**Affiliations:** 1 Department of Biology, University of New Mexico, Albuquerque, NM, United States; 2 Department of Mathematics and Statistics, University of New Mexico, Albuquerque, NM, United States; 3 The Mind Research Network, Albuquerque, NM, United States; 4 Department of Neurology, University of New Mexico, Albuquerque, NM, United States; 5 Center for Memory and Aging, University of New Mexico, Albuquerque, NM, United States; 6 Department of Molecular Genetics & Microbiology/Neurology, Albuquerque, NM, United States

**Keywords:** albumin index, ATN(V)I framework, blood–brain barrier, dynamic contrast-enhanced MRI, mixed dementia, neuroinflammation, permeability, vascular cognitive impairment

## Abstract

Inflammation and blood–brain barrier (BBB) disruption are increasingly implicated in cognitive decline, but it remains unclear which fluid and imaging measures best capture these processes. Participants were drawn from two overlapping research programs, MarkVCID and UNM ADRC. We studied 149 participants with Alzheimer’s disease, leukoaraiosis, mixed dementia, subcortical ischemic vascular dementia, or memory impairment. BBB permeability was assessed using the albumin index (Q_alb_), a global blood-to-CSF leakage ratio, and dynamic contrast-enhanced MRI permeability (K_trans_), a regional gadolinium transfer measure. Both were treated as permeability measures that inflammation may influence rather than as direct measures of inflammation. CSF and plasma biomarkers included matrix metalloproteinases, angiogenic factors, cytokines, GFAP, NfL, pTau181, and Aβ42/40; MRI measures included PSMD, mean free water, hippocampal volume, and cortical thickness. Univariate associations were screened using Spearman correlation and AIC, and multivariable models used AIC-based stepwise selection. Diagnosis-adjusted models, bootstrap selection frequencies, penalized regression, and cross-validated R^2^ were used as sensitivity analyses. Q_alb_ and K_trans_ were not significantly correlated (Spearman ρ = 0.14, p = 0.28), suggesting that they index different dimensions of BBB dysfunction without establishing biological independence. The multivariable CSF model for Q_alb_ retained MMP-2, Flt-1, IL-8, GFAP, and NfL and explained approximately half the variance (R^2^ = 0.53), whereas the plasma model explained less variance (R^2^ = 0.32). For K_trans_, multivariable CSF, plasma, and MRI models explained R^2^ = 0.36, 0.39, and 0.14, respectively, while PSMD was the strongest FDR-robust univariate MRI correlate. CSF–plasma correlations ranged from near zero for MMP-9 (r = −0.03) to stronger associations for NfL (r = 0.74) and IL-13 (r = 0.70), indicating that plasma cannot be assumed to substitute for CSF marker-by-marker. Q_alb_ and K_trans_ should be considered complementary rather than interchangeable measures. The Q_alb_–CSF model showed the strongest inflammation-related signal, but these findings are exploratory and require validation in independent, longitudinal cohorts before an inflammation axis can be incorporated into an ATN(V)-based framework.

## 1 Introduction

Alzheimer’s disease (AD) and related dementias (ADRD) are an increasing cause of neurological disease, mainly driven by the aging of populations worldwide (Snyder et al., 2015). As disease-modifying treatments (DMT) for dementia are introduced into clinical practice, it is becoming essential to more accurately diagnose patients that could benefit from those treatments and those that could have harmful side effects (Jack et al., 2016). Separating patients based on clinical evaluation has proven to be difficult because of the overlap of clinical features. This has resulted in the development of biomarkers to improve the classification of patients that are based on multiple modalities (Jack et al., 2024). Initial attempts at biological diagnoses with biomarkers were done in AD patients using levels of amyloid and tau in CSF and PET scans, resulting in the ATN formula, where A was amyloid, T was tau, and N was neurodegeneration. This early attempt failed to account for multiple etiologies, particularly the contribution of vascular disease, leaving out the large number of patients with mixed dementia due to the combination of AD and vascular pathology. With the approval of disease-modifying therapies (DMT), the identification of mixed pathology became more important because of the increase in side effects when vascular disease was present. We and others have expanded the research diagnostic formula by adding vascular disease (V) as determined from the detection of white matter ischemic injury on the diffusion tensor image (DTI) from MRI.

To expand the ATN formula to include vascular disease (V), we used a clustering classification method with two axes (the dichotomy method) or three axes (the trichotomy method) (Caprihan et al., 2021; Caprihan et al., 2023). The y-axis contained AD proteins, and the x-axis contained evidence derived from MRI of vascular disease. In the two-dimensional graph, the upper outer quadrant contained the mixed dementia patients (Caprihan et al., 2023). The growing importance of inflammation suggests (I) be added to the formula, since it contributes to progressive cognitive loss. The 2013 NIH Alzheimer’s Disease Summit broadened the research agenda beyond AD to include other causes of dementia, particularly the vascular contribution. This resulted in a seven-center consortium grant focused on developing validated biomarkers for use in clinical trials of vascular contributions to cognitive impairment and dementia, referred to as MarkVCID (Wilcock et al., 2021; Lu et al., 2021). The consortium selected biomarkers to study that could be used to define vascular dementia; they selected biomarkers from commercially available assay kits and from MRI that were available to all consortium members. Among the biomarkers were a number of inflammatory markers, including matrix metalloproteinases (MMP-3, MMP-9, and MMP-10), angiogenic factors (PlGF, VEGF), and pro-inflammatory cytokines (TNF-α, IL-1β, IL-6, IL-8, IL-13). The consortium subsequently validated peak-width of skeletonized mean diffusivity (PSMD), mean free water, and white-matter hyperintensity volume as neuroimaging biomarkers of small-vessel white-matter injury, all of which we use in the present analysis. Our group previously examined BBB permeability using Qalb and Ktrans and evaluated their relationships with inflammatory biomarkers. This report describes an initial attempt to identify potential biomarkers for an inflammation-related axis (I) that could be incorporated into the ATN(V) framework (Hillmer et al., 2023).

### 1.1 What Q_alb_ and K_trans_ do and do not measure

A clarification of terms is needed before the analyses that follow. Q_alb_ is a ratio of CSF to serum albumin and therefore indexes global blood-to-CSF barrier permeability; K_trans_ is a gadolinium transfer constant and therefore indexes regional microvascular permeability. Neither is a direct measure of inflammation. Inflammation is one of several processes that can increase barrier permeability, alongside endothelial ageing, small-vessel disease, altered transport, and tight-junction remodeling. Throughout this paper we therefore treat Q_alb_ and K_trans_ as permeability measures that inflammation may drive, and we use them as candidate read-outs against which fluid inflammatory markers can be evaluated not as inflammation markers in their own right.

This framing matters for interpretation. An association between a cytokine and K_trans_ does not establish that the two index the same underlying process. Inflammation and white-matter injury may arise together from shared upstream mechanisms, in particular chronic small-vessel disease, without either being a direct read-out of the other. Where such associations appear below, we consider that alternative explicitly rather than treating co-occurrence as evidence of a shared construct.

In this study, we address four questions: (1) Are Q_alb_ and K_trans_ correlated, or do they capture distinct aspects of BBB injury? (2) How do the CSF and plasma inflammatory biomarkers, matrix metalloproteinases (MMPs), angiogenic factors, and cytokines associate with the two measures? (3) How do MRI measures of white-matter microstructural injury relate to each BBB measure? (4) Can these results support the addition of an inflammation axis (“I”) to an expanded ATN(V) biomarker framework, yielding an ATN(V)I model of dementia? Together, these questions clarify which measurement modality (CSF, plasma, or MRI) carries the strongest inflammation-related signal associated with BBB permeability. The study is cross-sectional and exploratory: its purpose is to compare measurement compartments and generate hypotheses for an inflammation axis, not to validate one. The principal-component analyses are retained as secondary analyses and reported in the Supplement.

## 2 Materials and methods

### 2.1 Participants

The University of New Mexico Health Sciences Center Human Research Protections Office approved the study. All patients gave informed consent to study procedures, including lumbar puncture in a subset of participants. Patients were recruited from the community, clinics at the University of New Mexico Hospital including the Department of Neurology and the Center for Memory and Aging and the memory clinic at the Albuquerque Veterans Administration Hospital. All patients underwent neurological examinations, neuropsychological tests, venipuncture for blood, and an MRI; and a subgroup of consenting subjects had a lumbar puncture to collect CSF. Participant ages are summarized in Table 1. ApoE genotyping was not performed.

**TABLE 1:** Patient demographics by diagnosis. Values are median [Q1; Q3] for numeric variables, or n (%) for categorical.

Characteristic	N	Overall (N = 149)	AD (n = 26)	LA (n = 29)	Mixed (n = 22)	SIVD (n = 53)	MI (n = 19)	p-value
Age at assessment	149	68.0 [59.0; 75.0]	70.5 [65.0; 75.0]	63.0 [56.0; 70.0]	74.0 [68.0; 77.0]	67.0 [59.0; 74.0]	63.0 [48.0; 76.0]	0.005
Sex	149	​	​	​	​	​	​	0.026
Female	​	69 (46%)	13 (50%)	20 (69%)	7 (32%)	24 (45%)	5 (26%)	​
Male	​	80 (54%)	13 (50%)	9 (31%)	15 (68%)	29 (55%)	14 (74%)	​
(Q_alb_)	128	2.61 [2.23; 2.95]	2.61 [2.36; 2.94]	2.26 [1.88; 2.55]	2.74 [2.19; 2.91]	2.82 [2.53; 3.10]	2.54 [1.89; 2.71]	<0.001
(K_trans_)	73	2.21 [0.30; 3.34]	0.66 [0.07; 1.55]	1.59 [0.29; 2.45]	3.35 [1.72; 4.19]	3.25 [2.40; 4.25]	2.96 [−1.7; 3.3]	0.002

AD: Alzheimer’s disease; LA: leukoaraiosis; Mixed: mixed dementia; SIVD: subcortical ischemic vascular dementia; MI: memory-impairment comparison group. The p-value is for the omnibus test of a difference across all five diagnostic groups—Kruskal–Wallis for continuous variables and chi-square for sex. It does not refer to any single pairwise comparison; pairwise results are in Table 2.

Participants included 149 patients drawn from the overlapping MarkVCID and New Mexico Alzheimer’s Disease Research Center (NM ADRC) research programs. Patient diagnoses were assigned by consensus of three study neurologists into four diagnostic groups: Alzheimer’s disease (AD), subcortical ischemic vascular dementia (SIVD; within the vascular cognitive impairment and dementia [VCID] spectrum), mixed dementia (MX), and leukoaraiosis (LA); a small comparison group with memory impairment (MI) was also enrolled. AD diagnoses followed published criteria (McKhann et al., 2011); when available, we also used CSF amyloid and tau measurements. SIVD was diagnosed as described in a consensus paper (Rosenberg et al., 2016). Mixed dementia was diagnosed by combining the AD diagnosis, including CSF AD proteins when available, with white matter injury indicated by DTI. LA designated participants with significant white matter hyperintensities (WMHs) on FLAIR MRI but with minimal cognitive or neurological impairment. Patients with multiple or single strategic strokes were excluded. Because these groups differ substantially in the pathology driving barrier disruption, all primary models were additionally refit with diagnostic group as a covariate and, separately, after excluding AD-dominant cases (Section 3.6.2).

### 2.2 Blood and CSF studies

Two methods were used to measure BBB disruption: the albumin index (Q_alb_), defined as the ratio of CSF albumin to serum albumin, and dynamic contrast-enhanced MRI (DCE-MRI), from which we derived a regional permeability transfer constant (K_trans_). Throughout the manuscript, “Q_alb_” refers to the fluid-based ratio and “K_trans_” refers to the DCE-MRI-derived metric; we use this convention consistently to avoid confusion between the imaging modality (DCE-MRI) and the quantity it estimates (K_trans_). We use “Q_alb_” rather than “albumin index” throughout, including in figure axes and table headings.

#### 2.2.1 Phosphorylated tau and amyloid-β

Several biomarkers were measured in CSF and plasma. CSF biomarkers were obtained by lumbar puncture performed in the morning, generally after an overnight fast, by one of the authors (JCA). Blood draws were performed during the same patient visit. Samples were centrifuged, aliquoted, and stored at −80 °C for later analysis. Levels of CSF Tau protein phosphorylated at threonine 181 (pTau181) were measured using Innotest Phospho-Tau_181_ ELISA (Fujirebio US; Malvern, PA). In addition, we measured pTau217 by Simoa (Quanterix HD). All CSF underwent one freeze–thaw cycle prior to analysis. Assays were performed according to manufacturer protocols and read on a Bio-Tek multimodal plate reader with absorbance at 450 nm; concentrations were quantified from the manufacturer’s in-assay standard curve. We measured Aβ_1-42_ and Aβ_1-40_ to calculate the Aβ_1-42_/Aβ_1-40_ ratio (V-PLEX Aβ Peptide Panel 1 — 6E10; MesoScale Discovery, Rockville, MD). Concentrations were quantified using a 2-fold sample dilution and the supplied in-assay standard curve. All values are reported in pg/mL; the ratio is unitless.

#### 2.2.2 Matrix metalloproteinase, angiogenesis, and proinflammatory assays

As part of the MarkVCID consortium, we used MesoScale Discovery (MSD) multiplex assay kits to measure matrix metalloproteinases (MMP-1, MMP-2, MMP-3, MMP-9, and MMP-10) using two ELISA kits (MSD; MMP 2-Plex and MMP 3-Plex). Angiogenic growth factors (VEGF-C and placental growth factor, PlGF) were measured with the MSD Angiogenesis Panel 1. Pro-inflammatory cytokines (interleukins and tumor necrosis factor, TNF) were measured with the MSD Proinflammatory Panel 1. All CSF samples were run undiluted; plasma samples were diluted 2-fold, except for the MMP 3-Plex, where plasma was diluted 10-fold. All concentrations are reported in pg/mL.

Assays were performed using established protocols on an MSD Quickplex SQ 120 plate reader, with analysis in MSD Discovery Workbench 4.0 software. Marker concentrations were subjected to intra-plate variability tests using the coefficient of variation (CV) from duplicate runs of each sample; samples with CV ≥ 15% were removed from further analysis. In addition, two pooled CSF and two pooled plasma control samples were run in duplicate on every plate; the same intra-plate CV threshold (≥15%) was applied, and plate-to-plate variability was assessed against these controls.

### 2.3 MRI studies

To assess the integrity of the white matter, we used MRI scans acquired on a Siemens Prisma 3T scanner located at the Mind Research Network. Initial scans were performed with a 12-channel RF coil; later scans were acquired with a 32-channel RF coil. Imaging parameters were closely matched between coils. The 3D MPRAGE sequence had TR = 2,530 ms, four echoes, and TI = 1,200 ms with an acquisition time of 6.5 min. The 3D FLAIR sequence had TR = 6,000 ms, TE = 427 ms, and TI = 2000 ms. Diffusion data were collected with FOV = 224, 2 mm isotropic resolution, and 72 slices for both RF coils. On the 12-channel coil, a single-shell protocol with b = 800 s/mm^2^ (30 directions plus 5 b = 0 volumes) was used. On the 32-channel coil, three-shell multi-band diffusion data were collected; a single b = 800 s/mm^2^ or b = 1,000 s/mm^2^ shell with 55 directions was extracted for this study.

Validated MarkVCID neuroimaging kits were used for the calculation of white matter hyperintensities (WMH), peak width of skeletonized mean diffusivity (PSMD), and free water (Lu et al., 2021). WMH volumes were calculated from T1 and FLAIR images using a fully automated WMH detection and quantification pipeline (idealab.ucdavis.edu) adopted by the MarkVCID consortium. Diffusion images were corrected for motion and distortion; mean diffusivity (MD) and fractional anisotropy (FA) were calculated using FSL (www.fmrib.ox.ac.uk). PSMD and mean free water were calculated using the MarkVCID-consortium-adopted scripts (www.markvcid.org).

The blood–brain permeability MRI method has been described previously (Taheri et al., 2024). In brief, eight axial slices (4 mm thick, 2 mm in-plane resolution) covering a 3.2 cm imaging slab placed above the corpus callosum were imaged. Rapid inversion-recovery T1 measurements were made one before gadolinium contrast (Gd-DTPA, Magnevist) injection and eight afterwards, each separated by 2.5 min, for a total imaging time of 22.5 min, and used to derive K_trans_.

### 2.4 Cohort and biomarker availability

Because data were combined from two overlapping (NM ADRC and MarkVCID) studies, the number of participants with each biomarker varies. Table 3 summarizes biomarker availability by grant/source group and is the basis for the sample-size variations shown in subsequent analyses.

### 2.5 Statistical methods

#### 2.5.1 Data decisions

Numeric variables x_j_ were transformed as x_j_′ = log_2_ (x_j_ + k_j_) to address influential points caused by right skew, where k_j_ was typically the smallest strictly positive value; K_trans_ required a slightly larger k_j_ to avoid inducing left skew. We defined clinically plausible ranges for each assay; any value outside its range was set to missing before analysis. Each analysis used the subset of participants with complete data for the variables it required. Hypothesis tests used a two-sided type I error rate of α = 0.05. Primary exploratory p-values were unadjusted except for within-analysis multiple comparisons (e.g., Dunn’s *post hoc* tests with Holm correction); Benjamini–Hochberg FDR q-values for the univariate screens are reported as a sensitivity analysis below. For comparative analyses, BBB responses and continuous biomarker/MRI predictors were standardized (z-scored) where indicated; age was retained in years. However, when comparing the same proteins between CSF and Plasma, raw (unstandardized) values were used to maintain the original measurement scales and ensure more interpretable comparisons across biomarkers.

Multiplicity was handled as follows. Within-analysis multiple comparisons were adjusted (Dunn’s *post hoc* tests use the Holm correction). Elsewhere, unadjusted p-values are reported because the study is exploratory: its purpose is to establish which measurement compartment carries the strongest inflammation-related signal and to generate hypotheses, not to confirm pre-specified ones. Variable retention in the multivariable analyses was governed by an information criterion rather than by a p-value threshold, and model stability was further examined using bootstrap selection frequencies, penalized regression, and cross-validation. For transparency, we also report Benjamini–Hochberg false-discovery-rate q-values alongside the unadjusted p-values for every univariate association, computed within each response (Supplementary Table S2). Several of the strongest Q_alb_ associations and the PSMD–K_trans_ association remained significant after FDR correction.

### 2.6 Univariate associations of biomarkers with Q_alb_ and K_trans_

**FIGURE 1:**
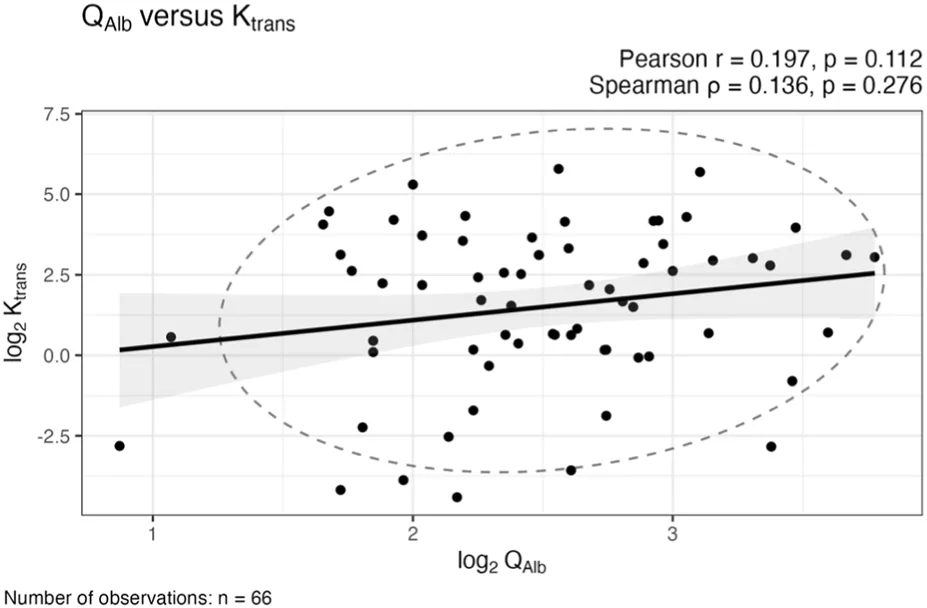
Scatterplot of Q_alb_ versus K_trans_ BBB permeability measures. Both axes are on the log_2_ scale, as used throughout the rest of the manuscript. Pearson and Spearman correlation coefficients with associated p-values for testing whether the correlations are different from zero are reported in the upper right. The solid line is the ordinary least-squares fit and the shaded band its 95% confidence region; the dashed ellipse is the 95% concentration ellipse of the bivariate distribution and is descriptive only.

Continuous demographic and permeability measures were compared across diagnostic groups using Kruskal–Wallis tests, followed where appropriate by Dunn’s *post hoc* tests with Holm correction. Sex distributions were compared using a chi-square test. The relationship between Q_alb_ and K_trans_ was assessed using Spearman’s rank correlation. This choice follows from the distributional properties of the two measures rather than from their shared interpretation. In the 66 participants with both measures, log_2_ Q_alb_ was consistent with normality (skewness −0.15; Shapiro–Wilk W = 0.989, p = 0.83), whereas log_2_ K_trans_ remained left-skewed and departed significantly from normality (skewness −0.61; W = 0.950, p = 0.010) (Supplementary Table S3). Given the non-normal distribution of K_trans_, a rank-based coefficient was used. Both Pearson and Spearman coefficients are reported in Figure 1 and agree in direction and non-significance, so the conclusion does not depend on the choice.

Each blood-brain barrier (BBB) response variable, albumin index (Q_alb_) and 3T white-matter permeability (K_trans_), was modeled as a function of each biomarker, age, and sex in a multiple regression model using stepwise model selection with the Akaike Information Criterion (AIC), with the main-effects model as the initial model and, where specified, allowing for biomarker-demographic interactions, to identify biomarkers retained in the models for each BBB response.

#### 2.6.1 Multivariate modeling of BBB permeability and fluid/MRI biomarkers

The relationships between BBB permeability (Q_alb_ and K_trans_) and fluid and MRI biomarkers were evaluated using multiple linear regression models. Separate models were fit for each combination of response variable and predictor biomarker group: CSF biomarkers, plasma biomarkers, and MRI measures.

Because data were combined from several studies with differences in data collection protocols, fluid biomarkers have structured patterns of missing data. To maximize the number of complete observations available for each analysis, we used a two-stage variable selection approach. First, candidate predictor variables were identified from preliminary univariate screening using Akaike Information Criterion (AIC) applied to individual predictor–response associations. Second, all possible subsets of the candidate variables were assessed for the number of participants with complete data across the response and all predictors in each subset. This step used visual inspection of missing data pattern plots, which display variable combinations sorted by the number of complete observations. We manually selected the variable subset that provided a balance between retaining as many biologically relevant predictors as possible and maintaining an adequate sample size for reliable estimation.

For the selected subset, a multiple regression model was fitted for each BBB permeability response using the chosen biomarkers plus age and sex as candidate predictors. Stepwise model selection was performed in both forward and backward directions using AIC, with the main-effects model serving as the initial model. For the Q_alb_–MRI, K_trans_–CSF, and K_trans_–MRI models, the upper search scope additionally allowed two-way interactions between each biomarker predictor and age or sex; the Q_alb_–CSF, Q_alb_–plasma, and K_trans_–plasma models were searched over main effects only. Model assumptions and observations with undue influence were assessed for the initial and final models.

Where interactions were allowed, biomarker × age and biomarker × sex terms were included in the search scope for stated biological reasons rather than as an unguided search. Barrier permeability increases with age independently of disease, so an age-dependent slope was considered plausible for biomarkers whose relationship with permeability may vary with age. Sex differences in immune response, cerebrovascular risk-factor burden, and reported Q_alb_ values have also been described, so a sex-dependent slope was likewise considered plausible. These interaction terms were therefore included as a biologically motivated class in the specified models rather than selected solely after inspecting individual results. Supplementary Table S2 reports the tested interaction terms with their unadjusted p-values and Benjamini–Hochberg q-values, allowing readers to assess the strength and multiplicity of these findings.

#### 2.6.2 Sensitivity and stability analyses

Three sets of sensitivity analyses were performed. First, because BBB measures differ across diagnostic categories (Table 2), each of the six models was refit with diagnostic group added as a covariate and, separately, after excluding AD-dominant cases (AD and mixed dementia). For the covariate-adjustment models, the diagnostic groups were collapsed into AD-dominant, vascular-dominant, and Other/MI categories to avoid estimating multiple diagnosis-specific parameters in models with limited complete-case sample sizes.

**TABLE 2:** Kruskal–Wallis test and post hoc pairwise comparisons by diagnosis for K_trans_ and Q_alb_. Only statistically significant pairwise differences are shown. Holm correction applied to Dunn’s post hoc tests. Abbreviations as in Table 1.

Measure	χ^2^	Df	p-value	Significant pairwise comparisons (dunn test, holm-adjusted)
MRI permeability (K_trans_, log_2_)	17.118	4	0.0018	AD–Mixed (p = 0.029), AD–SIVD (p = 0.006), LA–SIVD (p = 0.047)
Q_alb_ (log_2_)	18.707	4	<0.001	LA–SIVD (p = 0.0004)

Spearman correlation between K_trans_ and Q_alb_: ρ = 0.136, p = 0.276. The two measures are not significantly correlated.

Second, to assess how stable the stepwise selection was at these sample sizes, the entire selection procedure was repeated on 1,000 bootstrap resamples of each model’s complete-case data, and the proportion of resamples retaining each term was reported. An elastic-net model (α = 0.5, penalty chosen by ten-fold cross-validation) was fit to the same data as a penalized alternative to stepwise selection. Cross-validated R^2^ was estimated using 10-fold cross-validation repeated five times, with the AIC selection procedure repeated within each training fold. We report both the apparent and cross-validated R^2^ for every model to quantify model optimism.

Third, all univariate screens were also reported with Benjamini–Hochberg q-values as described in Section 3.5.1. Results of these analyses are reported in Supplementary Tables S2, S4, S5.

#### 2.6.3 Principal component analysis (PCA)

Another strategy to develop a composite score for evaluating inflammation-related BBB permeability is the use of principal component analysis (PCA). A subset of features is selected, and the first component (PC1), representing the direction of maximal variability, is regressed against Q_alb_; R^2^ is calculated, and linearity is assessed visually. Because PCA is unsupervised, PC1 captures the direction of maximal variance among the predictors rather than the direction most predictive of the response; therefore, it may underperform supervised regression in this setting, and we treat the PCA as a secondary comparison. These analyses are reported in Supplementary Section 6.4.

#### 2.6.4 CSF–plasma biomarker correlations

We examined the correlation between CSF and plasma concentrations of the same biomarker across individuals (for example, CSF pTau181 versus plasma pTau181). This analysis has two related purposes. First, it quantifies the extent to which CSF and plasma concentrations of the same biomarker covary, which is informative about the relationship between the central and peripheral measurements of each marker. Second, because lumbar puncture is invasive and not always available, it provides an initial indication of whether a plasma measurement might serve as a proxy for the corresponding CSF measurement. Pearson’s correlation coefficient (r) for each biomarker was calculated using complete CSF–plasma pairs. The full set of pairwise scatterplots is provided in the Supplement; an overall histogram of correlations and selected representative pairs are shown in Figure 10.

#### 2.6.5 Composite inflammation score and cognition

A composite inflammation-related score was constructed as a weighted sum of the standardized CSF biomarkers retained in the Q_alb_ -CSF model, using the corresponding regression coefficients as weights. Age and sex were not included in the composite so that the score represented the biomarker component of the model. MMSE and MoCA were not administered uniformly across the contributing cohorts and were therefore not available consistently for this sample. We instead examined domain T-scores for executive function, attention, memory, and language derived from the neuropsychological assessments available across the cohorts. Associations were evaluated using Spearman correlations and age- and sex-adjusted regression models.

## 3 Results

### 3.1 Demographics

The study included 149 patients (69 women, 80 men; Table 1). Diagnostic groups were AD (n = 26), LA (n = 29), Mixed (n = 22), SIVD (n = 53), and memory-impairment comparison group (MI) (n = 19). The LA group was mostly female; Mixed and MI were predominantly male; AD was approximately balanced. The median age varied between groups (Kruskal–Wallis p = 0.005): the youngest patients were LA (median 63, Q1–Q3: 56–70 years) and MI (63, 48–76), while the oldest were Mixed (74, 68–77).

The Kruskal–Wallis test revealed significant differences in both K_trans_ and Q_alb_ across diagnostic groups (K_trans_ χ^2^ = 17.12, p = 0.0018; Q_alb_ χ^2^ = 18.71, p < 0.001; Table 2). For Q_alb_ (log_2_), Dunn’s test showed values were lower in LA than in SIVD, with no other pairwise differences (Table 2). In contrast, K_trans_ was higher in SIVD than in AD and LA, and higher in Mixed than in AD; other comparisons, including those involving MI, were not significant. Biomarker availability varied across the three grant/source groups (MarkVCID, VCI, VDP): Q_alb_ was available in 128 of 149 participants, while K_trans_ was available in only 73, largely because the VDP cohort did not include DCE-MRI as part of its standard protocol (Table 3).

**TABLE 3:** Biomarker availability by grant/source group and grant identifier. The MarkVCID (UP), VCI, and VDP grants together contribute 149 unique patients; numbers show how many participants in each cohort had Q_alb_ and K_trans_ data available. Differences in K_trans_ availability across cohorts reflect study-specific MRI protocols (DCE-MRI was used most consistently in the MarkVCID and VCI cohorts).

Cohort	Grant Id	N	Q_alb_ (n available)	K_trans_ (n available)	Both Q_alb_ and K_trans_ (n)
MarkVCID	UP	49	45	34	31
VCI	VCI	39	36	35	32
VDP	VDP	61	47	4	3
Total	—	149	128	73	66

VDP, cohort: DCE-MRI, was not part of the standard protocol; only 4 participants have K_trans_ values.

### 3.2 Q_alb_ and K_trans_ capture different aspects of BBB permeability

Q_alb_ and K_trans_ were not significantly correlated (Spearman ρ = 0.14, p = 0.28; n = 66; Figure 1). We interpret this as suggesting that the two measures capture different dimensions of BBB dysfunction. It does not establish that they are biologically independent: the absence of a monotone association is equally compatible with partially overlapping mechanisms whose net effects do not covary across individuals, and the sample with both measures available is modest.

### 3.3 Top univariate correlates of Q_alb_ and K_trans_

**FIGURE 2:**
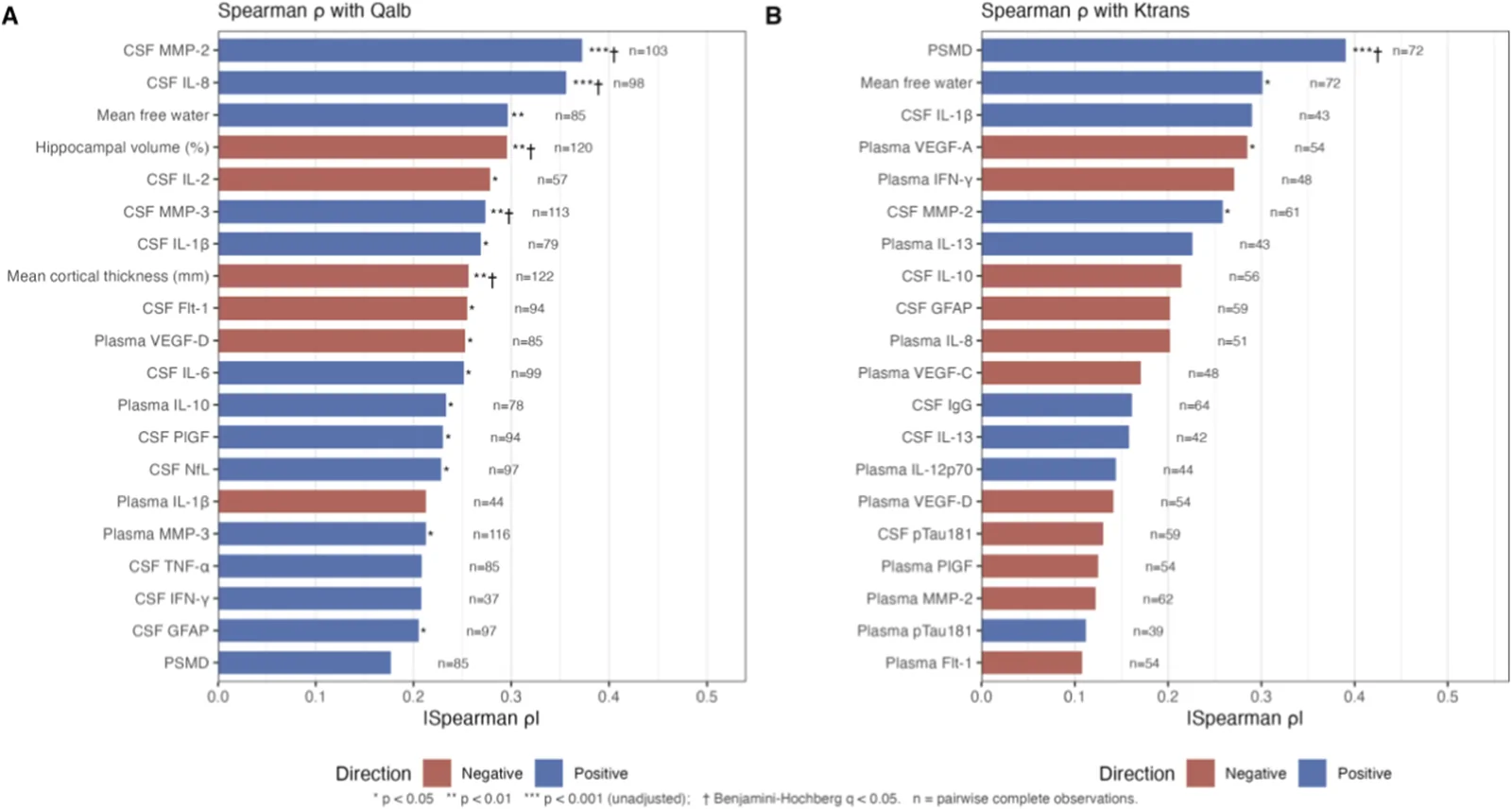
Top univariate correlates of Q_alb_ and K_trans_. Horizontal bar plots rank candidate fluid and MRI biomarkers by the absolute value of their Spearman rank correlation with each BBB response. **(A)** Predictors ranked by |Spearman ρ| with Q_alb_. **(B)** Predictors ranked by |Spearman ρ| with K_trans_. Bar length encodes |ρ| and bar fill color encodes the sign of the underlying correlation (blue = positive, red = negative). Sample sizes (n) shown to the right of each bar are pairwise complete observations between that predictor and the response. Significance stars indicate the p-value for testing ρ = 0 (*p < 0.05, **p < 0.01, ***p < 0.001, unadjusted). Abbreviations: CSF, cerebrospinal fluid; MMP, matrix metalloproteinase; IL, interleukin; TNF, tumor necrosis factor; GFAP, glial fibrillary acidic protein; NfL, neurofilament light; PlGF, placental growth factor; VEGF, vascular endothelial growth factor; PSMD, peak width of skeletonized mean diffusivity. A dagger marks associations that also survive Benjamini–Hochberg control at q < 0.05. CSF IgG and the IgG index are excluded from panel A because both rise with the same barrier leakage that raises CSF albumin, which makes their correlation with Q_alb_ close to circular.

Before formal model selection, we ranked candidate predictors by their univariate Spearman correlation with each BBB response (Figures 2A,B). For Q_alb_ (panel A), the strongest correlates were CSF MMP-2, CSF IL-8, MRI mean free water, and CSF MMP-3 (all positive), together with negative associations for hippocampal volume (%) and mean cortical thickness. For K_trans_ (panel B), the two strongest correlates were the MRI-derived measures DTI PSMD and mean free water (both positive), followed by CSF MMP-2 (positive). These exploratory rankings show prominent CSF associations with Q_alb_, whereas PSMD was the strongest FDR-robust univariate correlate of K_trans_. Benjamini–Hochberg q-values for every correlation in this figure are provided in Supplementary Table S2. Of the 56 biomarkers screened against Q_alb_, 16 had unadjusted p < 0.05 and 5 remained significant after false-discovery-rate correction: CSF MMP-2 (q = 0.006), CSF IL-8 (q = 0.009), hippocampal volume (q = 0.019), CSF MMP-3 (q = 0.048), and mean cortical thickness (q = 0.049). Of the 52 biomarkers screened against K_trans_, 4 had unadjusted p < 0.05, and PSMD remained significant after FDR correction (q = 0.037). These FDR-adjusted results are provided as a sensitivity analysis alongside the exploratory unadjusted associations.

### 3.4 Univariate associations of biomarkers with Q_alb_ and K_trans_

**FIGURE 3:**
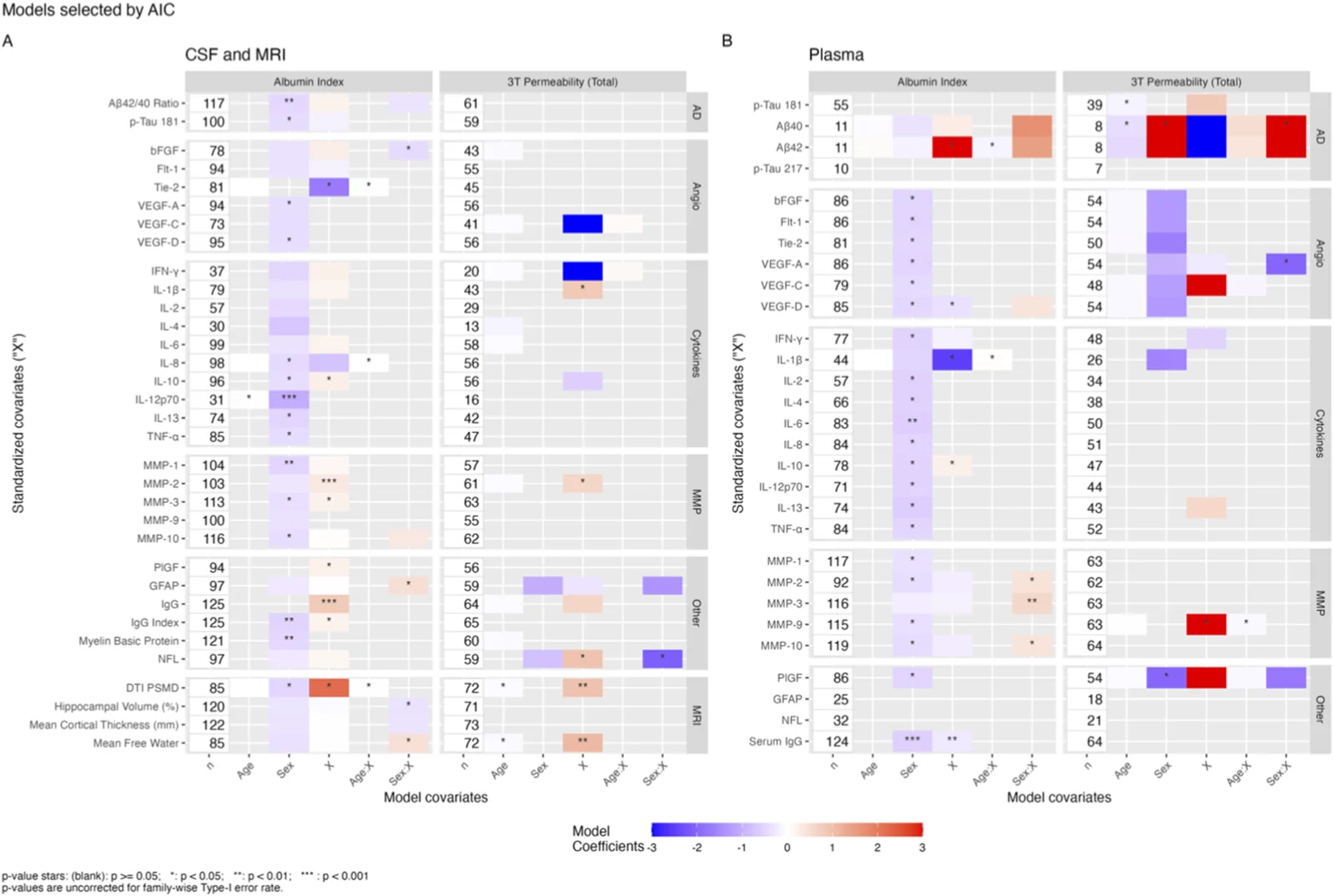
Univariate associations between biomarkers and BBB permeability. Each BBB response (Q_alb_ and K_trans_) appears as a facet column (top label). Biomarker groups are facet rows (right label), variables are aligned by biological category (AD-related, angiogenic factors, cytokines, MMPs, other markers, and MRI) so that the CSF/MRI side **(A)** and the plasma side **(B)** are directly comparable. Each biomarker in the separate multiple-regression models appears as a row (left label); the individual terms in the model are the columns (bottom labels), where “X” refers to the row-labelled biomarker. In the body, colored cells indicate variables retained after stepwise AIC selection; Gray cells indicate that the variable was not in the model. Star symbols are p-value categories (*p < 0.05, **p < 0.01, ***p < 0.001). Color encodes the sign of the standardized model coefficient (red positive, blue negative). The “n” column is the sample size for each regression. Colored cells without stars represent variables retained by AIC despite p ≥ 0.05; this is expected behavior of AIC selection. The full set of marginal scatter panels for each row of this figure is provided in the Supplement (Supplementary Figures S1-S3).

Q_alb_ was associated with 9 of the 29 CSF biomarkers examined, including inflammatory cytokines such as interleukin-6 (IL-6), interleukin-8 (IL-8), and tumor necrosis factor-α (TNF-α), as well as matrix metalloproteinases (MMPs) and angiogenic markers (Figure 3A). Q_alb_ was associated with only 3 of the 29 plasma biomarkers examined (Figure 3B), including amyloid beta 42 (Aβ42); these associations had relatively small effect sizes and were based on more restricted sample sizes, limiting interpretation. Potential explanations for the different CSF and plasma patterns are considered in the Discussion.

Sex was retained as a covariate in several Q_alb_ models. Biomarker × age and biomarker × sex interactions were examined as biologically motivated interaction classes as described in the Methods. Interpretation of sex-related findings and the relevant literature is considered in the Discussion rather than here.

#### 3.4.1 K_trans_ associations

Among CSF biomarkers, no retained main biomarker effect reached p < 0.05 in the separate Figure 3 models (Figure 3A, right column). Several panels showed age- or sex-moderated patterns, including CSF IgG, CSF MMP-2, and CSF VEGF-C, but these were driven by interactions rather than independent main effects and should be interpreted cautiously. Among plasma biomarkers (Figure 3B, right column), plasma VEGF-A showed a sex-moderated association with K_trans_ (sex × predictor interaction p = 0.033). Among MRI metrics, DTI PSMD (β = 0.934, 95% CI [0.307, 1.56], p = 0.004) and mean free water (β = 1.08, 95% CI [0.402, 1.75], p = 0.002) showed strong positive associations with K_trans_; age was negatively associated in both models.

### 3.5 Multiple regression analysis

We fit six multiple-regression models, one for each combination of BBB response (Q_alb_, K_trans_) and predictor block (CSF biomarkers, plasma biomarkers, MRI measures). For each model the sample size (n), the number of retained covariates (k), and the variance explained (R^2^) are summarized in Table 4; full coefficient estimates are reported in the corresponding subsections. Across all six models, Q_alb_ had the highest apparent R^2^ with CSF biomarkers (R^2^ = 0.53), followed by plasma biomarkers (R^2^ = 0.32) and MRI measures (R^2^ = 0.24). For K_trans_, apparent R^2^ was 0.36 with CSF biomarkers, 0.39 with plasma biomarkers (n = 35) and 0.14 with MRI measures, the last retaining only mean free water and age. The CSF/Q_alb_ model is the most informative single model in this paper and is the benchmark we return to in the Discussion. These are apparent R^2^ values; cross-validated R^2^ values, which provide a more realistic assessment of out-of-sample performance, are reported in Table 4 and Supplementary Table S5.

**TABLE 4:** Summary of the six multiple-regression models. For each combination of BBB response variable and predictor block, the table shows the number of participants with complete data (n), the number of covariates retained after stepwise AIC selection (k), and the proportion of variance explained. A cross-validated R^2^ column was obtained by repeating the entire selection procedure inside each training fold.

Response	Predictor block	n	k retained	R^2^	R^2^ (cross-validated)
Q_alb_	CSF biomarkers	54	5	0.53	0.19
Q_alb_	Plasma biomarkers	58	5 (incl. sex)	0.32	−0.04
Q_alb_	MRI measures	82	5 (incl. sex)	0.24	−0.09
K_trans_	CSF biomarkers	50	5 (incl. sex and sex × GFAP)	0.36	−0.13
K_trans_	Plasma biomarkers	35	4	0.39	0.02
K_trans_	MRI measures	70	2 (age, free water)	0.14	−0.11

#### 3.5.1 Q_alb_ ∼ CSF biomarkers

Q_alb_ was modeled as a function of CSF biomarkers using a broad set of 13 candidate CSF predictors identified through univariate AIC screening. After reviewing missing-data patterns (Supplementary Table S1), we selected markers of amyloid and tau pathology (Aβ42/40 ratio, p-Tau), angiogenic factors (Flt-1), cytokines (IL-6, IL-8, IL-10), matrix metalloproteinases (MMP-1, MMP-2, MMP-3, MMP-10), PlGF, GFAP, and NfL. This subset provided 54 complete observations.

**FIGURE 4:**
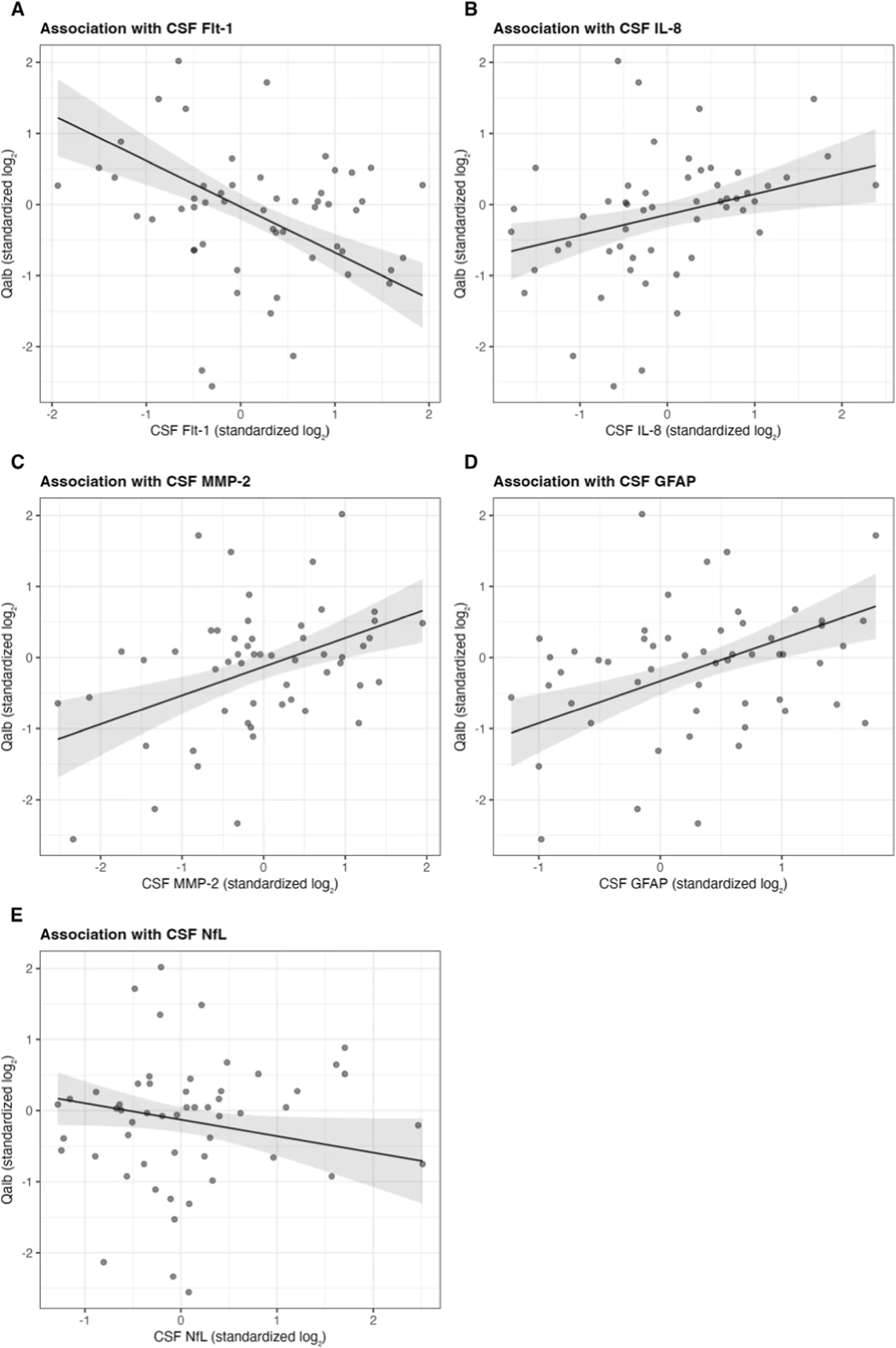
Q_alb_ and selected CSF biomarkers, multiple regression. Panels show regression plots from the final stepwise model (N = 54). **(A)** CSF Flt-1. **(B)** CSF IL-8. **(C)** CSF MMP-2. **(D)** CSF GFAP. **(E)** CSF NfL. Shaded regions indicate 95% confidence bands. Axes are on standardized (z-score) units.

The model had the highest apparent R^2^ among the six models (F (5, 48) = 10.96, p < 0.001, R^2^ = 0.53), explaining approximately half the variance in Q_alb_. The final model retained five CSF predictors: Flt-1, IL-8, MMP-2, GFAP, and NfL. Age and sex were not selected. CSF Flt-1 had the strongest individual association (β = −0.65, 95% CI [−0.89, −0.40], p < 0.001; Figure 4A). CSF IL-8 was positively associated (β = 0.29, 95% CI [0.09, 0.49], p = 0.006; Figure 4B). CSF MMP-2 was positively associated (β = 0.40, 95% CI [0.20, 0.61], p < 0.001; Figure 4C). CSF GFAP was positively associated (β = 0.59, 95% CI [0.31, 0.88], p < 0.001; Figure 4D). CSF NfL had a negative coefficient that did not reach the p < 0.05 threshold (β = −0.23, 95% CI [−0.47, 0.01], p = 0.055; Figure 4E).

#### 3.5.2 Q_alb_ ∼ plasma biomarkers

To model Q_alb_ as a function of plasma biomarkers, candidate predictors were selected from univariate AIC screening. After reviewing missing-data patterns (Supplementary Table S1), we selected seven plasma variables, yielding 58 complete observations: VEGF-D, IL-1β, IL-10, MMP-2, MMP-3, MMP-10, and serum IgG.

**FIGURE 5:**
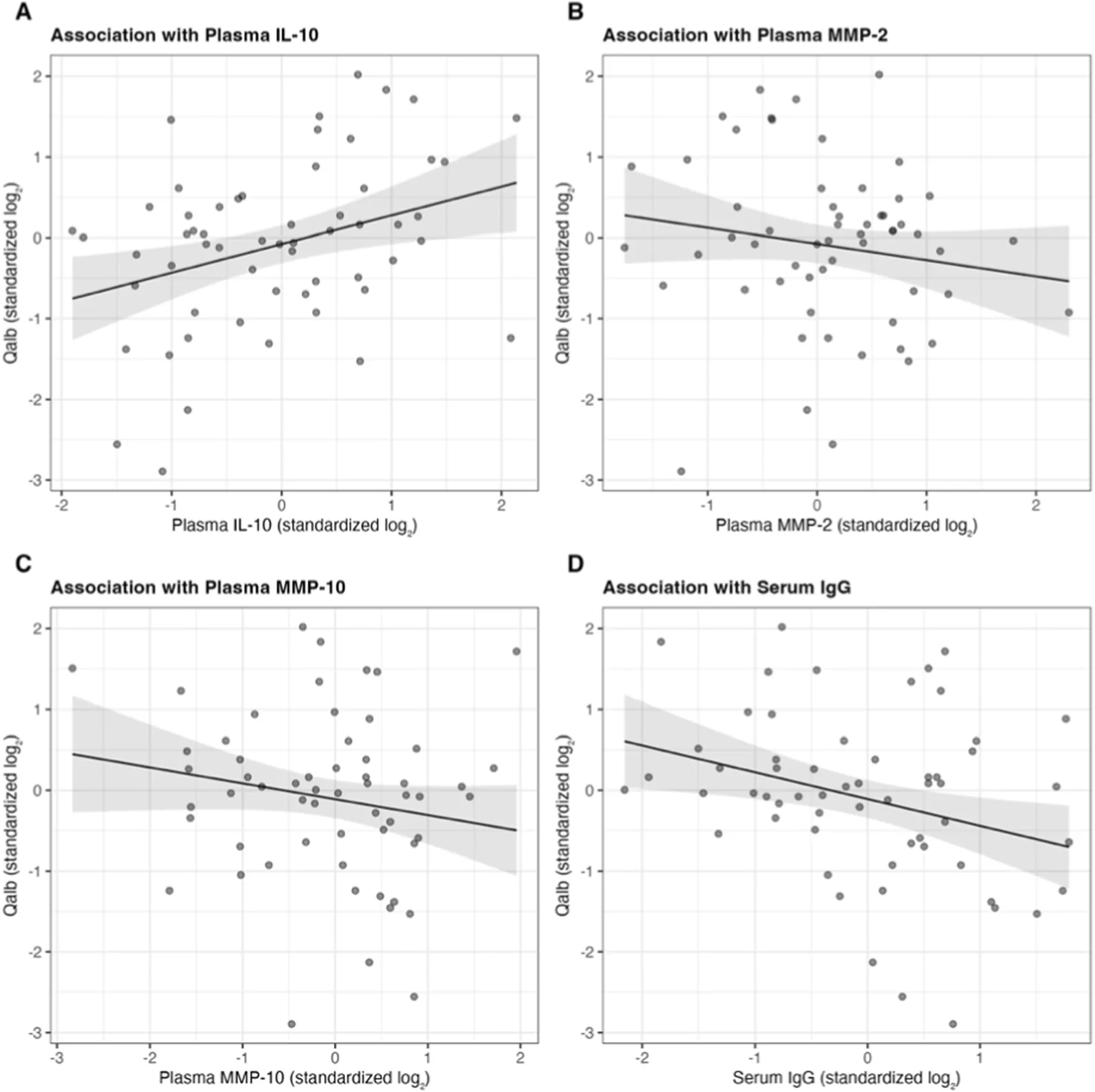
Q_alb_ and plasma biomarkers. Panels show regression plots from the final stepwise model (N = 58). **(A)** Plasma IL-10. **(B)** Plasma MMP-2. **(C)** Plasma MMP-10. **(D)** Serum IgG. Shaded regions indicate 95% confidence bands.

The selected model retained sex, plasma IL-10, MMP-2, MMP-10, and serum IgG (F (5, 52) = 4.97, p < 0.001, R^2^ = 0.32). Plasma IL-10 was positively associated with Q_alb_ (β = 0.36, 95% CI [0.10, 0.61], p = 0.007; Figure 5A). MMP-2 (β = −0.20, p = 0.176; Figure 5B) and Plasma MMP-10 (β = −0.20, p = 0.118; Figure 5C) were retained by AIC but were not significant at the 0.05 level. Serum IgG showed a negative association (β = −0.33, 95% CI [−0.58, −0.08], p = 0.010; Figure 5D). Sex was retained in the model, with males showing a higher estimated Q_alb_ than females (Male–Female contrast = 0.49, p = 0.048).

#### 3.5.3 Q_alb_ ∼ MRI measures

To assess whether structural white-matter and cortical measures relate to BBB integrity, we examined four MRI predictors: DTI PSMD, mean free water fraction, hippocampal volume (%), and mean cortical thickness. All 82 participants had complete data for these variables plus Q_alb_ (Supplementary Table S1).

**FIGURE 6:**
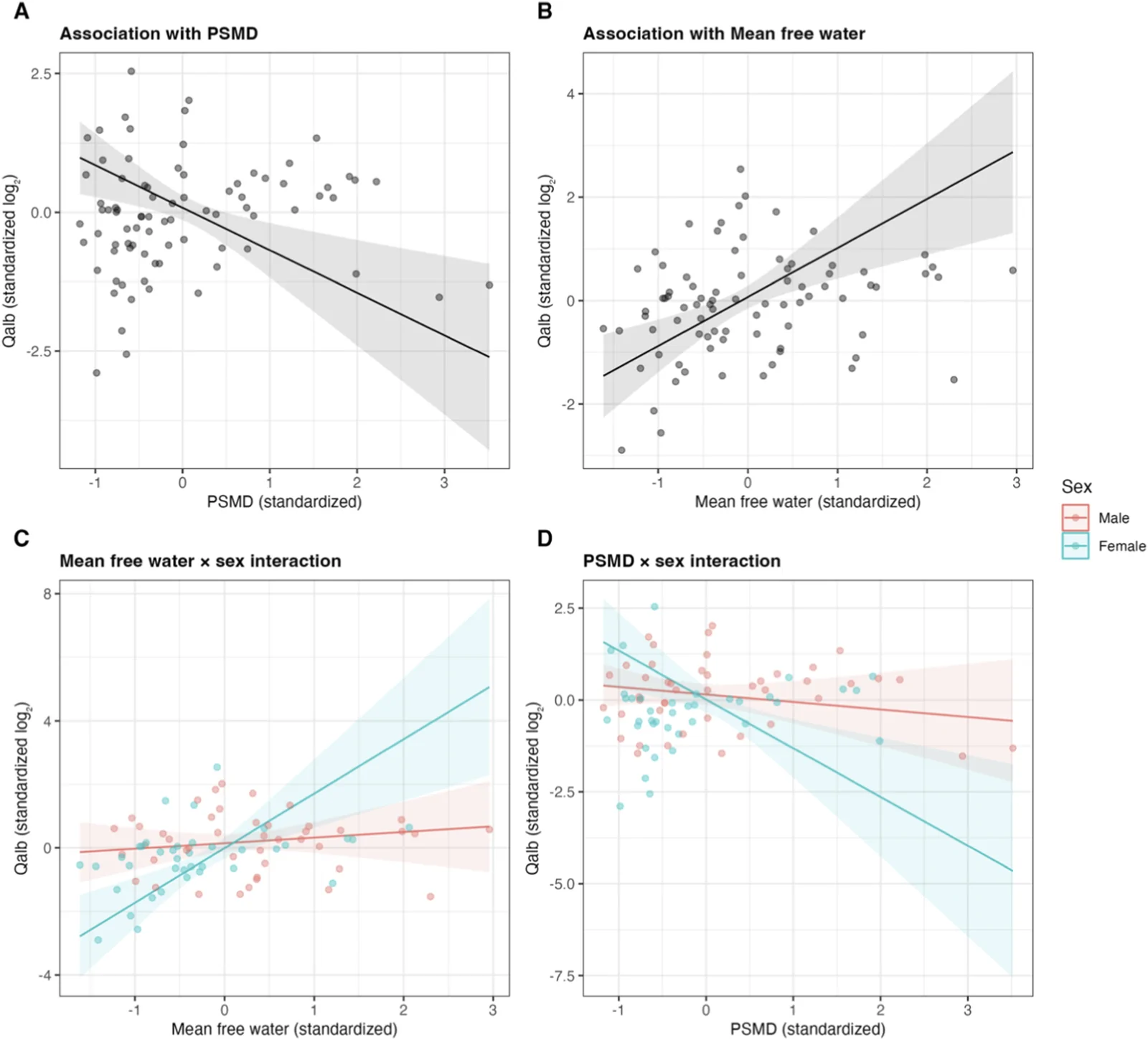
Q_alb_ and structural MRI measures. Panels show regression plots from the final stepwise model (N = 82). **(A)** Main effect of DTI PSMD. **(B)** Main effect of mean free water. **(C)** Interaction of sex with mean free water, with separate regression lines for males (red) and females (blue). **(D)** Interaction of sex with DTI PSMD. Shaded regions indicate 95% confidence bands.

The selected model retained sex, DTI PSMD, mean free water, and two interaction terms (sex × free water and sex × PSMD; F (5, 76) = 4.72, p < 0.001, R^2^ = 0.24). Hippocampal volume and cortical thickness were not retained. The pooled association between DTI PSMD and Q_alb_ was negative (β = −0.77, 95% CI [−1.25, −0.28]; Figure 6A), and between free water and Q_alb_ was positive (β = 0.95, 95% CI [0.44, 1.45]; Figure 6B). Both effects were modified by sex. For free water, the slope was essentially flat in males (β = 0.18, 95% CI [−0.33, 0.68]) but steeply positive in females (β = 1.72, 95% CI [0.83, 2.60]; slope contrast p = 0.004; Figure 6C). For DTI PSMD, the slope was near zero in males (β = −0.20, 95% CI [−0.66, 0.25]) but negative in females (β = −1.33, 95% CI [−2.18, −0.47]; slope contrast p = 0.024; Figure 6D). Sex modified both MRI associations, although in opposite directions: free water was positively associated with Q_alb_ in females, whereas PSMD showed a negative association in females; both slopes were near zero in males.

#### 3.5.4 K_trans_ ∼ CSF biomarkers

To examine the relationship between K_trans_ and CSF biomarkers, we selected a subset of CSF variables from prior univariate AIC screening. After evaluating missing-data patterns (Supplementary Table S1), we chose a subset providing 50 participants with complete data across the response, three CSF predictors (GFAP, IgG, NfL), plus age and sex.

**FIGURE 7:**
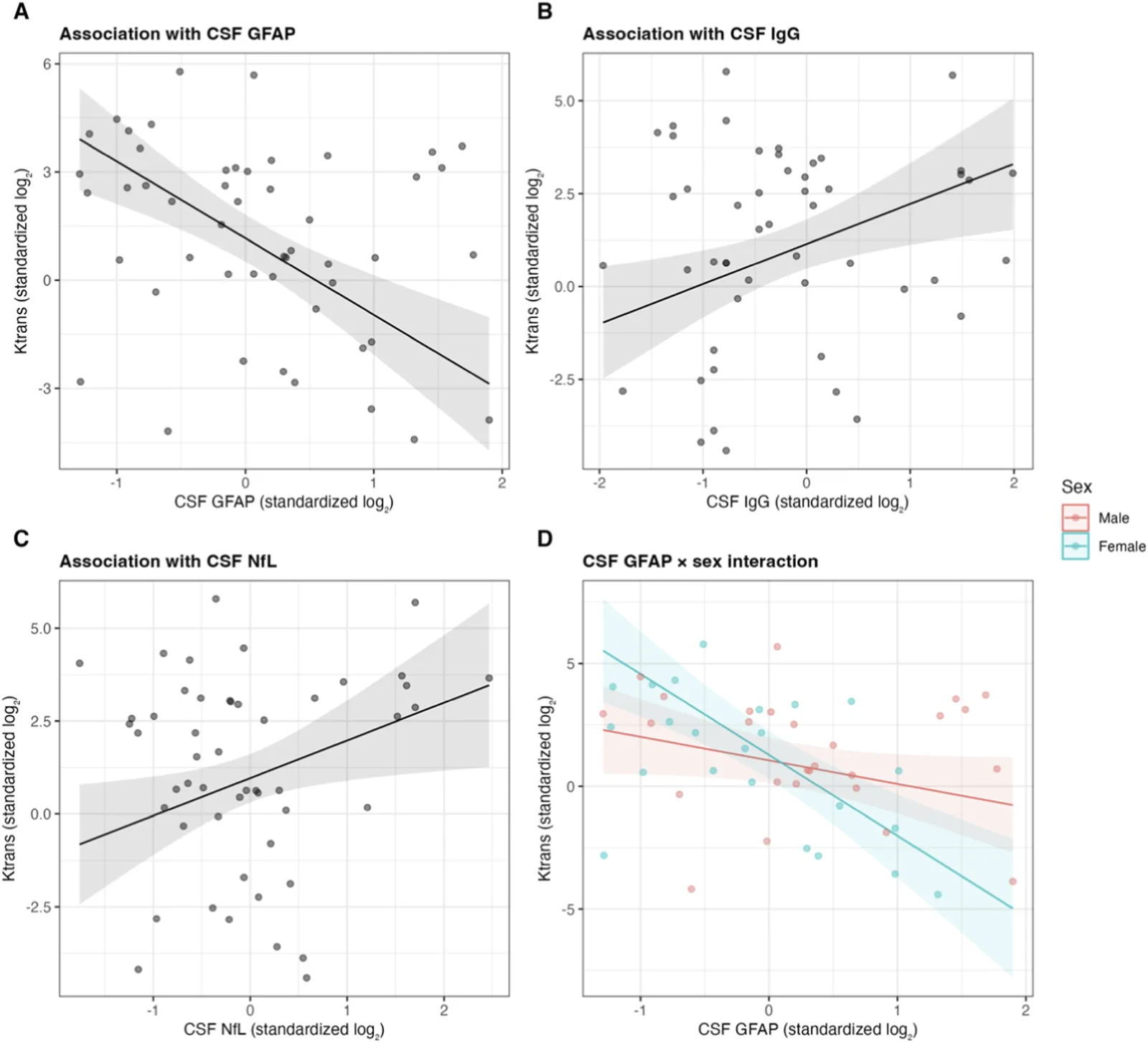
K_trans_ and CSF biomarkers. Panels show regression plots from the final stepwise model (N = 50). **(A)** Main effect of CSF GFAP. **(B)** Main effect of CSF IgG. **(C)** Main effect of CSF NfL. **(D)** Interaction of sex with CSF GFAP, with separate regression lines and 95% confidence bands for males (red) and females (blue).

The selected model retained sex, CSF GFAP, CSF IgG, CSF NfL, and the interaction between sex and CSF GFAP (F (5, 44) = 5.05, p < 0.001, R^2^ = 0.36). The main effect of CSF GFAP is shown in Figure 7A. CSF IgG was positively associated with K_trans_ (β = 1.08, 95% CI [0.32, 1.84], p = 0.007; Figure 7B). CSF NfL was also positively associated (β = 1.01, 95% CI [0.17, 1.86], p = 0.020; Figure 7C). The most striking finding was the significant sex × GFAP interaction (p = 0.005; Figure 7D): in females, higher CSF GFAP was associated with lower K_trans_ (β = −3.30, 95% CI [−4.71, −1.88]), whereas in males the association was weaker (β = −0.96, 95% CI [−1.98, 0.06]). The difference in slopes between males and females was 2.34 (p = 0.005). The main effect of sex on K_trans_ was not significant (Male estimated mean = 0.95, 95% CI [−0.10, 2.00]; Female = 0.91, 95% CI [−0.22, 2.05]; contrast p = 0.96). With n = 50 and an interaction term in the selected model, this finding should be regarded as provisional; bootstrap selection frequencies for this term are reported in Supplementary Table S5.

#### 3.5.5 K_trans_ ∼ plasma biomarkers

The relationship between K_trans_ and plasma biomarkers was examined using six candidate plasma variables. Missing-data evaluation (Supplementary Table S1) identified a subset with 35 complete observations across the response and six plasma predictors (VEGF-A, VEGF-C, IFN-γ, IL-13, MMP-9, PlGF).

**FIGURE 8:**
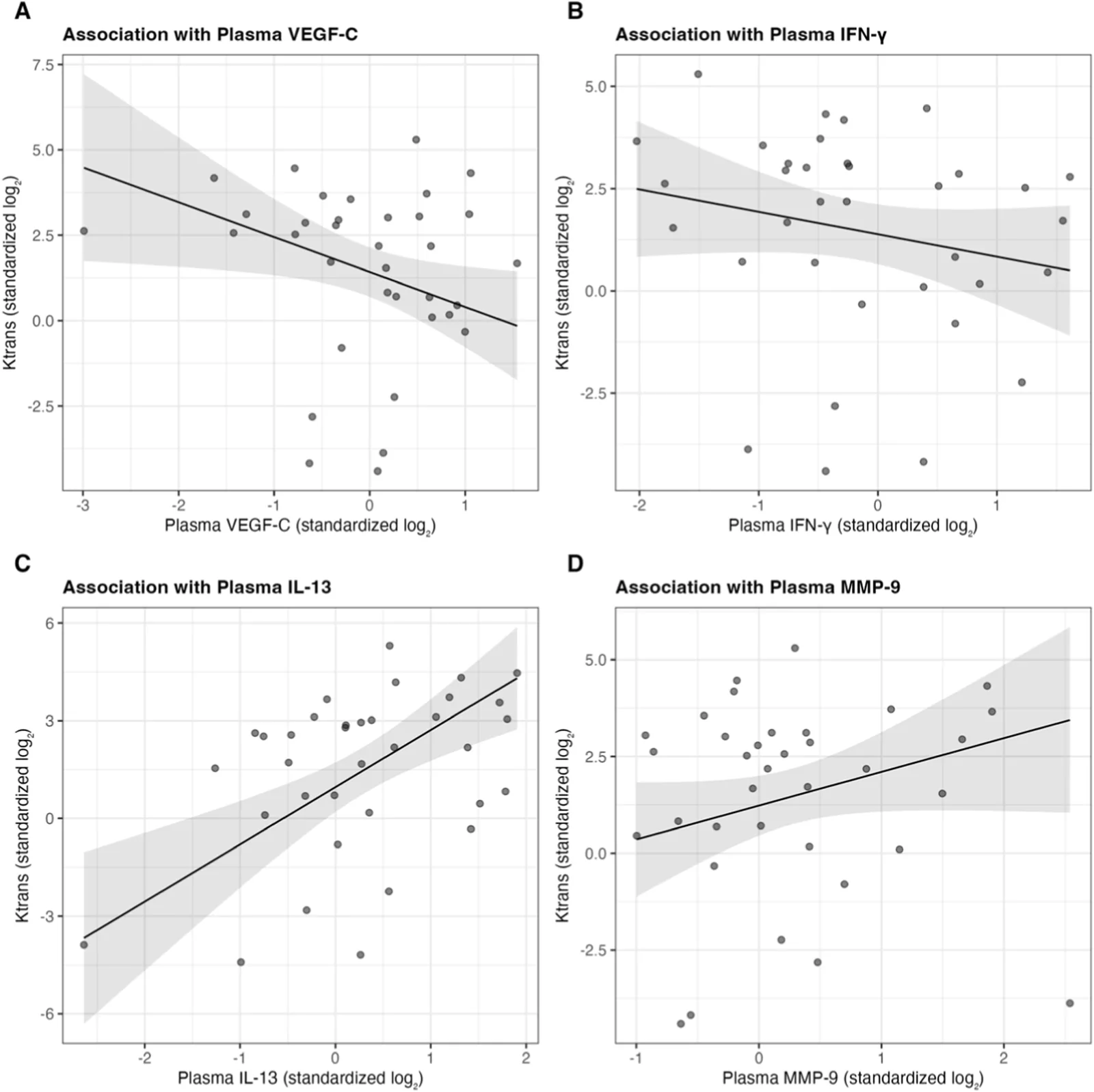
K_trans_ and plasma biomarkers. Panels show regression plots from the final stepwise model (N = 35). **(A)** Plasma VEGF-C. **(B)** Plasma IFN-γ. **(C)** Plasma IL-13. **(D)** Plasma MMP-9. Shaded regions indicate 95% confidence bands.

The selected model retained plasma VEGF-C, IFN-γ, IL-13, and MMP-9 (F (4, 30) = 4.89, p = 0.004, R^2^ = 0.39). Age and sex were not retained. Plasma VEGF-C showed a significant negative association (β = −1.02, 95% CI [−1.92, −0.13], p = 0.027; Figure 8A). Plasma IFN-γ showed a non-significant negative coefficient that did not reach p < 0.05 (β = −0.55, 95% CI [−1.35, 0.25], p = 0.173; Figure 8B). Plasma IL-13 was the strongest predictor, with a positive association with K_trans_ (β = 1.76, 95% CI [0.89, 2.62], p < 0.001; Figure 8C), and plasma MMP-9 showed a positive coefficient that did not reach p < 0.05 (β = 0.87, 95% CI [−0.14, 1.89], p = 0.088; Figure 8D). Although R^2^ is comparable to the Q_alb_–CSF model, the small n = 35 means estimates are imprecise and should be interpreted with caution.

#### 3.5.6 K_trans_ ∼ MRI measures

The four MRI measures (DTI PSMD, mean free water fraction, hippocampal volume (%), mean cortical thickness) were examined as predictors of K_trans_; 70 participants had complete data (Supplementary Table S1).

**FIGURE 9:**
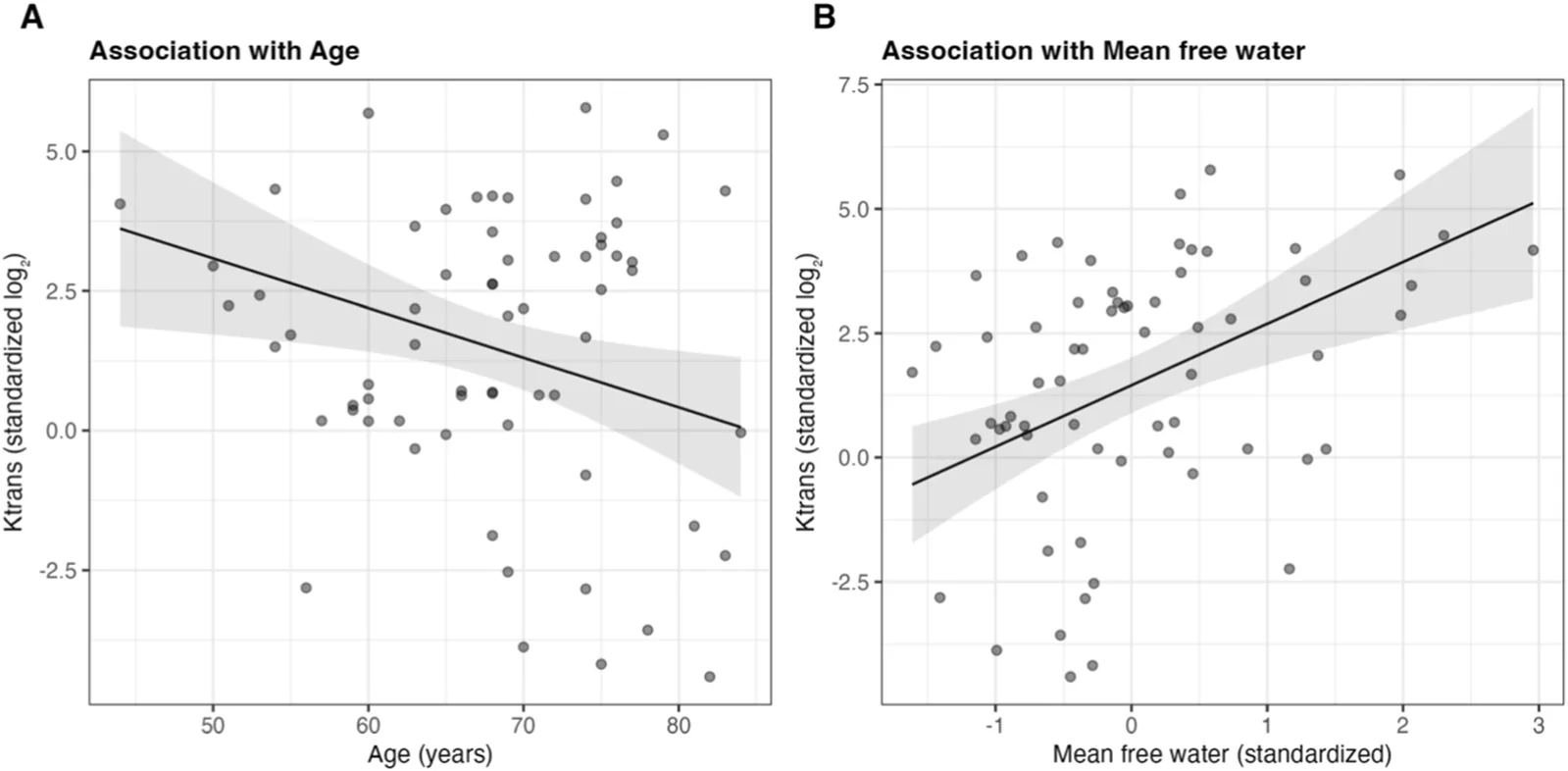
K_trans_ and structural MRI measures. Panels show regression plots from the final stepwise model (N = 70). **(A)** Age at assessment. **(B)** Mean free water. Shaded regions indicate 95% confidence bands.

The selected model retained only age and mean free water (F (2, 67) = 5.67, p = 0.005, R^2^ = 0.14). Sex, DTI PSMD, hippocampal volume, and cortical thickness were all dropped. Age showed a small but significant negative association (β = −0.08, 95% CI [−0.15, −0.01], p = 0.026; Figure 9A). Mean free water was positively associated with K_trans_ (β = 1.08, 95% CI [0.40, 1.76], p = 0.002; Figure 9B). The model explained a modest proportion of variance, indicating that structural MRI features alone capture only part of the variability in K_trans_. Among the candidate structural MRI measures, only mean free water was retained alongside age. Mean free water is a marker of localized white-matter injury, which is consistent with K_trans_ carrying a regional, injury-related component; the CSF and plasma models for K_trans_ had higher apparent R^2^, however, so this should not be read as showing that K_trans_ is solely an imaging-derived injury measure.

### 3.6 Sensitivity and stability analyses

Adjusting for diagnostic group did not materially improve five of the six reported models. Adding the collapsed diagnosis variable to each final model gave p = 0.080 for Q_alb_–CSF, p = 0.480 for Q_alb_–plasma, p = 0.569 for Q_alb_–MRI, p = 0.799 for K_trans_–CSF, and p = 0.944 for K_trans_–MRI. The exception was the K_trans_–plasma model (p = 0.029), which had the smallest sample size (n = 35) and uneven representation across diagnostic groups; this result is therefore interpreted cautiously. Excluding AD-dominant cases reduced the available samples to 19–42 participants per model and produced generally similar patterns, although estimates from these smaller samples were less stable and their R^2^ values should be considered descriptive (Supplementary Table S4). Overall, these analyses suggest that the Q_alb_ associations are not readily explained by diagnostic composition.

Model stability was evaluated using three complementary approaches (Supplementary Tables S5a-5c). First, the complete stepwise-selection procedure was repeated in 1,000 bootstrap resamples. The Q_alb_–CSF model showed particularly high selection frequencies for MMP-2 (99%), Flt-1 (97%), GFAP (92%), IL-8 (85%), and NfL (63%). Several terms in the other models were also frequently selected, although stability was less consistent across predictor blocks. Second, elastic-net regression (α = 0.5, with the penalty selected by ten-fold cross-validation) was used as a penalized alternative to stepwise selection. At the conservative one-standard-error penalty, only the Q_alb_–CSF model retained predictors (Flt-1, IL-8, IL-10, MMP-2, and GFAP), four of which were also retained in the reported AIC model. Third, cross-validated R^2^, obtained by repeating model selection within each training fold, was 0.19 for Q_alb_–CSF and 0.02 for K_trans_–plasma and was negative for the remaining four models, compared with apparent R^2^ values of 0.53, 0.32, 0.24, 0.36, 0.39, and 0.14, respectively. Taken together, these analyses indicate that the Q_alb_–CSF model has the strongest and only appreciable out-of-sample performance, whereas the remaining models show substantially greater apparent than cross-validated performance. These findings reinforce the exploratory nature of the analyses and the need for validation in independent cohorts.

### 3.7 CSF–plasma biomarker correlations

**FIGURE 10:**
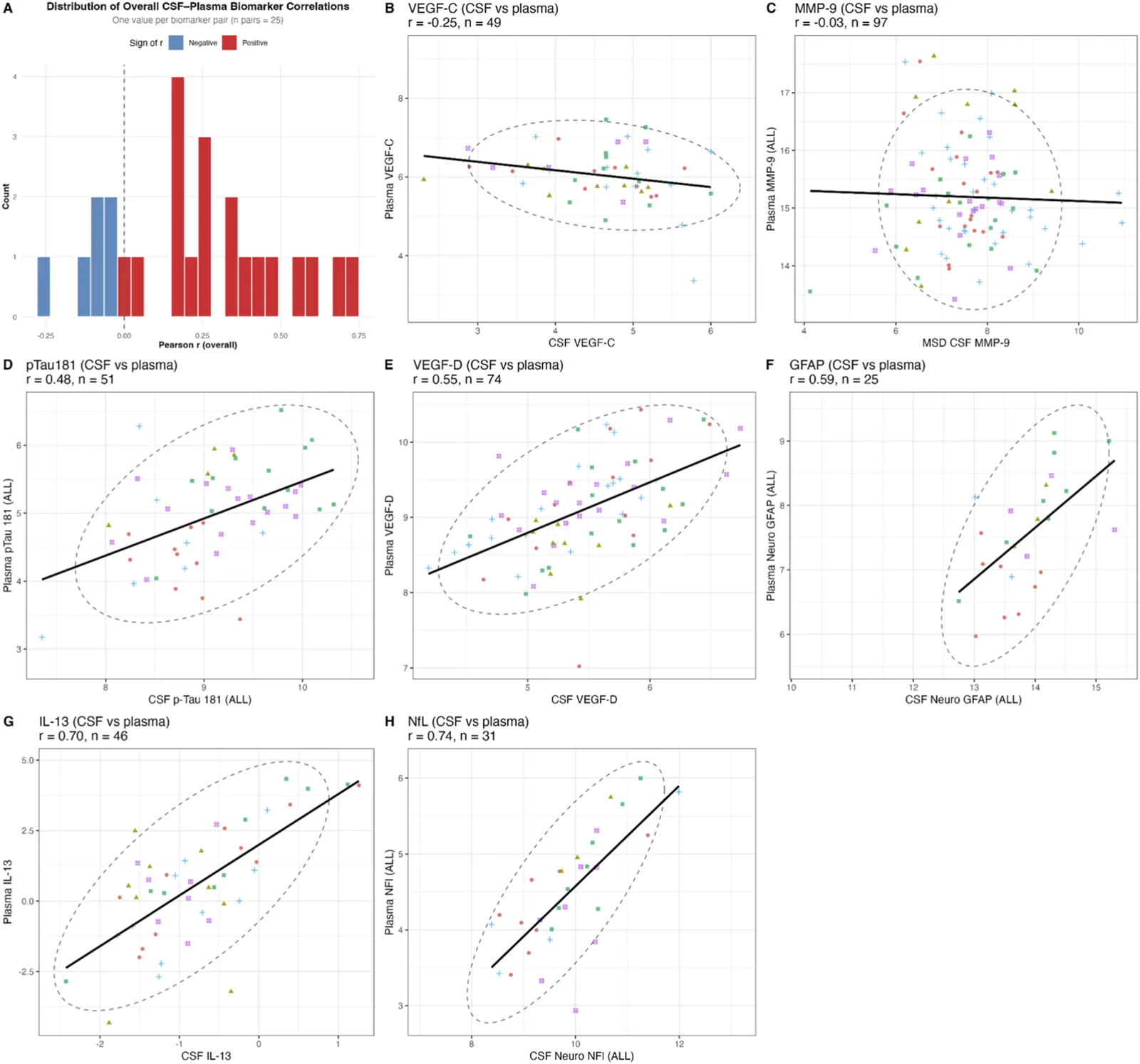
CSF–plasma correlations within each biomarker. **(A)** Histogram of correlations across all biomarker pairs (one value per pair). **(B–H)** A selected set of biomarkers showing the range of patterns, from near-zero (MMP-9) to moderately positive (NfL, IL-13). r-values are Pearson correlation coefficients; n is the number of complete CSF–plasma pairs. Point color indicates diagnostic group for visualization only and does not affect the correlation analysis.

Across the cohort, CSF–plasma correlations varied across biomarkers rather than following one consistent pattern (Figure 10A). The distribution shows that most correlations were modest, and the strongest observed correlation was ∼0.75, with no biomarkers exceeding that level in this dataset. While Figure 10A summarizes the correlation (one value per CSF–plasma biomarker pair), panels B–H highlight a subset of representative CSF–plasma relationships to illustrate the range of patterns.

VEGF-C showed a weak negative association between compartments (Figure 10B; r = −0.25), and MMP-9 demonstrated essentially no cross-fluid coupling (Figure 10C; r = −0.03). Moving toward positive associations, pTau181 showed a moderate positive relationship (Figure 10D; r = 0.48), followed by VEGF-D (Figure 10E; r = 0.55) and GFAP (Figure 10F; r = 0.59). The strongest CSF–plasma correlation was seen for IL-13 (Figure 10G; r = 0.70) and NfL (Figure 10H; r = 0.74). Overall, the figure emphasizes that CSF–plasma coupling can range from weakly negative to moderately strong positive, depending on the marker, and that plasma cannot be assumed to substitute for CSF across the board.

### 3.8 Composite inflammation score and cognition

The exploratory composite inflammation-related score was not significantly associated with any cognitive domain after correction for multiple comparisons or after adjustment for age and sex (Supplementary Table S6).

## 4 Discussion

### 4.1 Summary of findings

We set out to determine whether two measures of blood–brain barrier permeability, Q_alb_ and K_trans_, capture the same phenomenon; which of CSF, plasma, or MRI carries the most information about each; and whether the resulting picture supports consideration of an inflammation axis within the ATN(V) framework. Four findings follow. First, Q_alb_ and K_trans_ were not significantly correlated, suggesting that they capture different dimensions of barrier dysfunction rather than interchangeable measures of the same process. Second, the Q_alb_–CSF model showed the strongest overall and cross-validated performance, whereas K_trans_ showed associations across CSF, plasma, and MRI predictor blocks with limited out-of-sample performance. Third, CSF and plasma concentrations of the same protein were variably correlated, indicating that plasma cannot be assumed to substitute for CSF marker-by-marker. Fourth, sex modified some of the observed relationships, most notably the association between white-matter free water and Q_alb_. Taken together, these findings support the presence of an inflammation-related biomarker signal associated with BBB permeability while emphasizing that its measurement and clinical interpretation remain uncertain.

### 4.2 Q_alb_ and K_trans_ index different dimensions of barrier dysfunction

Q_alb_ reflects the movement of albumin, a 66 kDa protein, from blood into CSF, and because it is computed from paired blood and CSF samples it represents a global signal of barrier leakage across the neuraxis. K_trans_ measures the transfer of a much smaller gadolinium chelate across small vessels within a defined white-matter region. The absence of correlation between them, which is consistent with our earlier report (Hillmer et al., 2023), suggests that they measure different dimensions of barrier dysfunction: a global, protein-scale, fluid-based leakage signal on the one hand and a regional, microvascular permeability signal on the other (Rosenberg, 2022).

A null correlation is not evidence of biological independence. Two partially overlapping processes may fail to covary across individuals because of differing time courses, anatomical coverage, underlying disease mechanisms, or measurement error in either measure. The practical implication is therefore not that Q_alb_ and K_trans_ are biologically unrelated, but that they should be considered complementary rather than interchangeable measures of BBB permeability. Where both are available, they may provide different information about barrier dysfunction.

**FIGURE 11:**
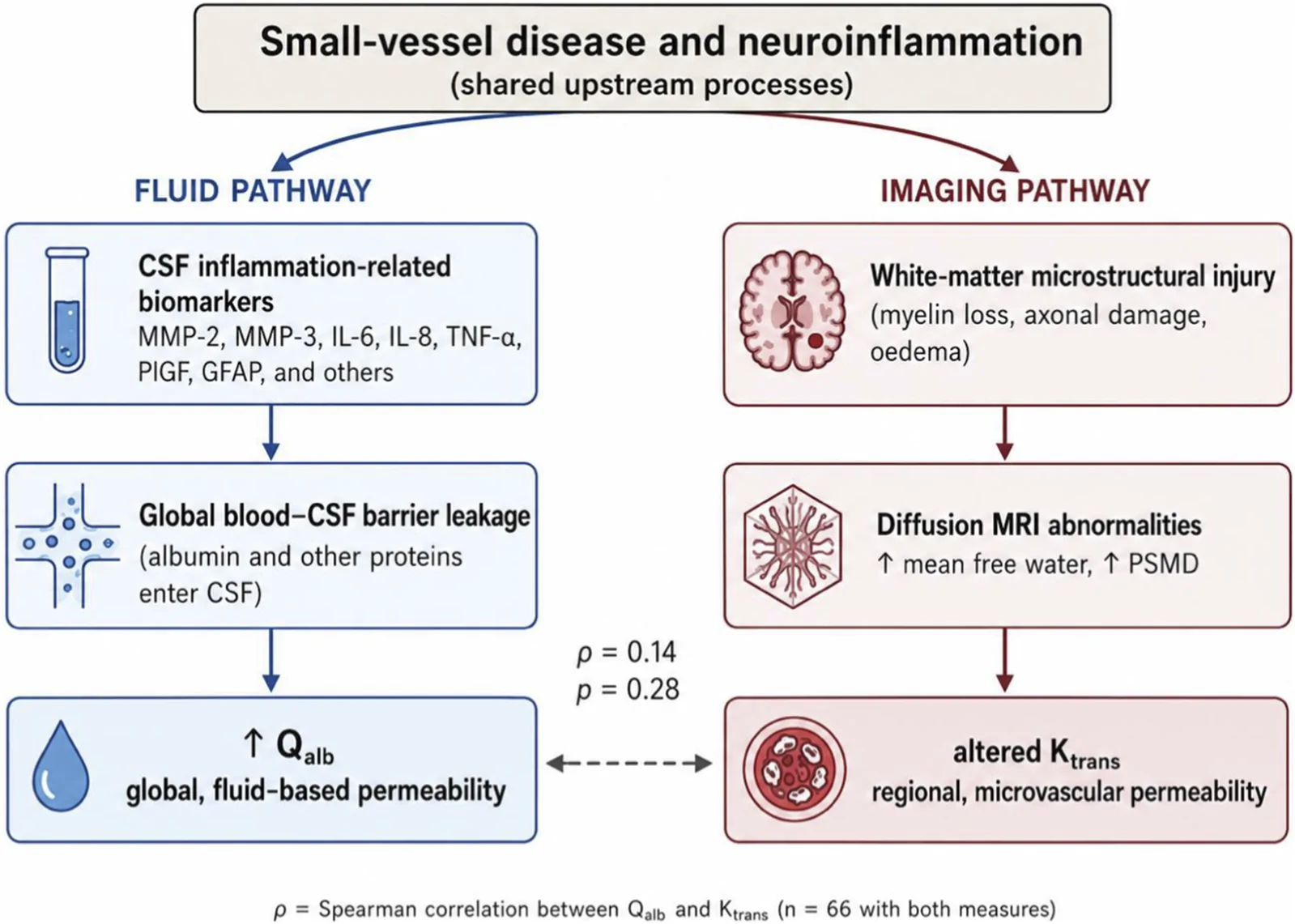
Conceptual model of the two pathways examined in this study. Left: CSF inflammatory biomarkers are associated with global blood–CSF barrier leakage and hence with elevated Q_alb_. Right: white-matter microstructural injury is reflected in diffusion MRI abnormalities and altered K_trans_. The two permeability measures were not significantly correlated in this cohort. Small-vessel disease and neuroinflammation may contribute to both pathways, so co-occurrence of inflammatory and imaging abnormalities does not by itself establish that they represent the same underlying construct.

The relationship between K_trans_ and inflammatory markers deserves the same caution. Associations between inflammatory biomarkers and K_trans_ do not necessarily mean that K_trans_ is a direct read-out of inflammation. Chronic small-vessel disease may contribute to both endothelial inflammation and white-matter microstructural injury, allowing the two to co-occur through shared upstream mechanisms without either being a direct measure of the other. Figure 11 illustrates these alternative pathways. Distinguishing among them will require longitudinal data, which the present cross-sectional design cannot provide.

### 4.3 What the CSF associations do and do not show

Several CSF biomarkers showed exploratory univariate associations with Q_alb_, including matrix metalloproteinases, inflammatory cytokines, and angiogenic factors. Among these, MMP-2 and IL-8 were also retained in the multivariable Q_alb_–CSF model, together with Flt-1, GFAP, and NfL. The biological functions represented by these biomarkers are coherent with processes that may accompany BBB dysfunction. Matrix metalloproteinases can degrade components of the basal lamina and tight junctions (Candelario-Jalil et al., 2009) and are expressed in cells located near the neurovascular unit, including astrocytes, microglia, and macrophages (Rosenberg et al., 2001). Angiogenic factors regulate vascular growth and remodeling, and newly formed or remodeling vessels may exhibit altered permeability (Greenberg and Jin, 2005). Cytokines can exert both pro- and anti-inflammatory effects, which is one reason the direction of individual associations should not be interpreted in isolation.

GFAP and NfL require particularly cautious interpretation. GFAP is an indicator of astrocytic activation rather than a specific measure of inflammation. Reactive astrogliosis can occur in response to inflammation but also to ischemia, trauma, and neurodegeneration. NfL reflects neuroaxonal injury and is elevated across a broad range of neurological conditions. Their association with Q_alb_ is therefore better interpreted as evidence that barrier leakage co-occurs with glial activation and axonal injury than as evidence that either marker is itself an inflammatory mediator.

The negative NfL coefficient in the multivariable Q_alb_ model, despite a positive univariate association, provides an additional reason for caution. This change in direction may reflect collinearity or suppression among correlated predictors in the relatively small complete-case sample rather than a direct inverse biological relationship. Individual regression coefficients in the multivariable models should therefore be interpreted within the context of the other retained predictors.

### 4.4 CSF versus plasma

CSF and plasma concentrations of the same protein were often only weakly related, ranging from essentially no association for MMP-9 to substantially stronger correlations for NfL and IL-13. Even for NfL, a correlation of approximately 0.74 corresponds to only about 55% shared variance between the two compartments. Several mechanisms may contribute to this incomplete correspondence. Peripheral production can contribute substantially to plasma concentrations and dilute their relationship with CNS concentrations; hepatic and renal clearance differ between compartments; proteins differ in stability in blood and CSF; and transport across the BBB is selective. Assay-level variability may add further noise, particularly for proteins present at low concentrations in plasma.

The balanced conclusion is not that plasma is uninformative. For markers such as NfL and IL-13, plasma concentrations track the corresponding CSF concentrations to a meaningful extent, and plasma IL-13 was among the stronger plasma associations with K_trans_ in our data. Plasma also has major practical advantages: it can be obtained repeatedly, is more acceptable to patients, and is substantially more scalable than lumbar puncture. These advantages have already made blood-based biomarkers increasingly important in dementia research and clinical practice (Palmqvist et al., 2025; Quispialaya et al., 2025).

What our results argue against is the assumption that plasma can substitute for CSF marker-by-marker without validation. A more realistic approach is to identify the specific inflammatory or injury-related biomarkers for which CSF–plasma coupling is sufficiently strong and reproducible, and to develop sensitive assays for those markers rather than attempting to translate an entire CSF biomarker panel directly into blood.

### 4.5 Sex differences

Sex was associated with several relationships in this cohort. Males had a higher estimated Q_alb_ than females in the plasma model, which is consistent in direction with prior reports. Wallin and colleagues found higher Q_alb_ values in male than female patients with vascular dementia (Wallin et al., 2000), and Algotsson and Winblad reported greater barrier compromise in male than female patients with AD (Algotsson and Winblad, 2007). The consistency in direction with prior studies provides external support for the observation, although replication in independent samples remains necessary. These findings suggest that sex should be considered in the development and interpretation of future Q_alb_-based composite measures. Sex-related differences have also been reported in Alzheimer’s disease-related tau pathology, including greater tau aggregation in females (Robinson et al., 2026).

A more notable finding was the interaction between sex and white-matter free water in the Q_alb_ model. In females, the slope relating free water to Q_alb_ was positive, whereas the corresponding association in males was substantially weaker. The PSMD interaction also differed by sex, but in the opposite direction: the association was negative in females and near zero in males. These observations raise the possibility that the relationship between white-matter microstructural injury and BBB leakage may differ by sex.

Possible explanations include sex-related differences in endothelial physiology, immune response, and cerebrovascular risk profiles, but these mechanisms were not measured directly in this study and remain speculative.

These findings should nevertheless be interpreted as hypothesis-generating. The free-water interaction arose in a sample of 82 participants and was part of a broader biologically motivated class of age- and sex-interaction tests rather than a single pre-registered hypothesis. In the broader interaction sensitivity analysis, nominal interaction findings did not survive false-discovery-rate correction. Replication in independent cohorts is therefore required before sex-specific relationships between permeability and white-matter injury can be considered established.

### 4.6 Implications for an ATN(V)I framework

Biomarker frameworks for dementia have expanded from amyloid and tau alone to include neurodegeneration and, increasingly, other pathological processes such as vascular disease. Mixed Alzheimer and cerebrovascular pathology is common in dementia (Schneider et al., 2007; Toledo et al., 2013; Snowdon et al., 1997) and the vascular contribution has additional clinical relevance in the era of anti-amyloid therapies, in which vascular abnormalities may influence the risk of amyloid-related imaging abnormalities. Neuroinflammation is therefore a plausible additional dimension to consider within an expanded biomarker framework.

Our results speak to that proposal in a specific and limited way. They identify a CSF biomarker pattern associated with Q_alb_ that showed the strongest and most reproducible performance among the models examined, although its cross-validated performance was substantially lower than its apparent fit. Plasma measurements reflected the corresponding CSF biomarkers only variably, and K_trans_ showed a different pattern of associations across fluid and imaging measures.

These findings do not demonstrate that adding an inflammation axis improves diagnosis, prognosis, or treatment selection beyond existing ATN(V)-based approaches. The study is cross-sectional and exploratory and was not designed to evaluate incremental clinical value. We therefore view the present results as providing a hypothesis-generating basis for considering an inflammation-related axis and an initial benchmark against which future biomarkers can be evaluated.

Establishing ATN(V)I as a useful framework will require validation in independent cohorts, longitudinal data linking inflammation-related biomarkers to disease progression and cognitive trajectories, and blood-based assays capable of reproducing clinically meaningful information obtained from CSF. Nothing in the present data supports current clinical application of an ATN(V)I framework.

### 4.7 Strengths and limitations

A principal strength of this study is that Q_alb_ and K_trans_ were measured in overlapping participants, which is uncommon because the two measures are generally studied separately. Their direct comparison was possible because of the lumbar-puncture and imaging protocols used in the contributing studies. The cohort also spans a range of clinical phenotypes, including leukoaraiosis with minimal cognitive or neurological impairment, Alzheimer’s disease, mixed dementia, and subcortical ischemic vascular dementia, with diagnoses assigned through neurological evaluation. The biomarker panel is broad, and the imaging measures include established MarkVCID-derived measures of vascular and white-matter injury (Maillard et al., 2022). In earlier work, white-matter ischemic injury was assessed with magnetic resonance spectroscopy, in which a reduced N-acetylaspartate signal was the most informative marker (Brooks et al., 1997; Sappey-Marinier et al., 1992). Diffusion tensor imaging has since replaced it here because it is more widely available.

The limitations are substantial and interrelated. First, the study is cross-sectional, so the temporal ordering of inflammation, BBB permeability, and white-matter injury cannot be determined. Associations between biomarkers and permeability therefore should not be interpreted as evidence of causality.

Second, participants were recruited at a single center, and the high prevalence of white-matter disease reflects the referral patterns and research focus of that site. The observed biomarker–permeability relationships may therefore not generalize to cohorts with different demographic or diagnostic compositions.

Third, complete-case sample sizes differed substantially across analyses because biomarker and imaging availability varied across the contributing studies. Missingness was therefore partly design-driven, and sample sizes for the six multivariable models ranged from 35 to 82 participants. Sensitivity analyses excluding AD-dominant cases were correspondingly smaller and should be interpreted cautiously.

Fourth, stepwise model selection can be unstable at these sample sizes. We therefore evaluated model stability using bootstrap selection frequencies, elastic-net regression, and cross-validated R^2^. The Q_alb_–CSF model showed the strongest stability and the highest cross-validated performance, but its cross-validated R^2^ remained substantially lower than its apparent R^2^. The remaining models showed little or no out-of-sample predictive performance. Apparent R^2^ therefore describes fit to the observed sample, whereas cross-validated R^2^ provides a more realistic assessment of expected performance in new observations.

Fifth, fluid biomarker concentrations can vary with fasting state, time of day, sleep, recent infection, stress, medication use, and systemic inflammatory state, whereas only single measurements were available in this study. The biomarker panel was also selected according to the assays available through the contributing studies and does not capture the full range of inflammatory proteins measurable with newer high-dimensional platforms (Ashton et al., 2025).

Finally, ApoE genotype was not available for the present analyses, and vascular risk factors were not modeled individually. Residual confounding by genetic, vascular, metabolic, and other clinical factors therefore cannot be excluded.

### 4.8 Conclusion

Q_alb_ and K_trans_ should be treated as complementary rather than interchangeable measures of BBB permeability. Among the models examined, the Q_alb_–CSF model showed the strongest and most reproducible inflammation-related biomarker signal, whereas plasma associations were more limited and marker-specific and K_trans_ showed a different pattern across fluid and imaging measures. These findings provide preliminary support for considering an inflammation-related axis within future ATN(V)-based frameworks, but they do not establish such an axis or demonstrate clinical utility. Validation in independent, longitudinal, multicenter cohorts is the necessary next step.

## Data Availability

The raw data supporting the conclusions of this article will be made available by the authors on reasonable request, subject to the data-sharing requirements and Institutional Review Board approval of the University of New Mexico.

## References

[B1] Algotsson A. Winblad B. (2007). The integrity of the blood-brain barrier in alzheimer's disease. Acta Neurol. Scand. 115, 403–408. 10.1111/j.1600-0404.2007.00823.x 17511849

[B2] Ashton N. J. Benedet A. L. Molfetta G. D. Pola I. Anastasi F. Fernandez-Lebrero A. (2025). Biomarker discovery in Alzheimer's and neurodegenerative diseases using nucleic acid linked immuno-sandwich assay. Alzheimers Dement. 21, e14621. 10.1002/alz.14621 40401628 PMC12096316

[B3] Brooks W. M. Wesley M. H. Kodituwakku P. W. Garry P. J. Rosenberg G. A. (1997). 1H-MRS differentiates white matter hyperintensities in subcortical arteriosclerotic encephalopathy from those in normal elderly. Stroke 28, 1940–1943. 10.1161/01.str.28.10.1940 9341699

[B4] Candelario-Jalil E. Yang Y. Rosenberg G. A. (2009). Diverse roles of matrix metalloproteinases and tissue inhibitors of metalloproteinases in neuroinflammation and cerebral ischemia. Neuroscience 158, 983–994. 10.1016/j.neuroscience.2008.06.025 18621108 PMC3584171

[B5] Caprihan A. Raja R. Hillmer L. J. Erhardt E. B. Prestopnik J. Thompson J. (2021). A double-dichotomy clustering of dual pathology dementia patients. Cereb. Circ. Cogn. Behav. 2, 100011. 10.1016/j.cccb.2021.100011 34746872 PMC8570532

[B6] Caprihan A. Hillmer L. Erhardt E. B. Adair J. C. Knoefel J. E. Prestopnik J. (2023). For alzheimer's disease neuroimaging I. A trichotomy method for defining homogeneous subgroups in a dementia population. Ann. Clin. Transl. Neurol. 10, 1802–1815. 10.1002/acn3.51869 37602520 PMC10578887

[B7] Greenberg D. A. Jin K. (2005). From angiogenesis to neuropathology. Nature 438, 954–959. 10.1038/nature04481 16355213

[B8] Hillmer L. Erhardt E. B. Caprihan A. Adair J. C. Knoefel J. E. Prestopnik J. (2023). Blood-brain barrier disruption measured by Albumin index correlates with inflammatory fluid biomarkers. J. Cereb Blood Flow Metab 43, 712–721. 10.1177/0271678X221146127 36522849 PMC10108191

[B9] Jack C. R. Jr. Bennett D. A. Blennow K. Carrillo M. C. Feldman H. H. Frisoni G. B. (2016). A/T/N: an unbiased descriptive classification scheme for alzheimer disease biomarkers. Neurology 87, 539–547. 10.1212/WNL.0000000000002923 27371494 PMC4970664

[B10] Jack C. R. Jr. Andrews J. S. Beach T. G. Buracchio T. Dunn B. Graf A. (2024). Revised criteria for diagnosis and staging of alzheimer's disease: alzheimer's association workgroup. Alzheimers Dement. 20, 5143–5169. 10.1002/alz.13859 38934362 PMC11350039

[B11] Lu H. Kashani A. H. Arfanakis K. Caprihan A. DeCarli C. Gold B. T. (2021). MarkVCID cerebral small vessel consortium: II. Neuroimaging protocols. Alzheimers Dement. 17, 716–725. 10.1002/alz.12216 33480157 PMC8627001

[B12] Maillard P. Hillmer L. J. Lu H. Arfanakis K. Gold B. T. Bauer C. E. (2022). MRI free water as a biomarker for cognitive performance: validation in the MarkVCID consortium. Alzheimers Dement. (Amst) 14, e12362. 10.1002/dad2.12362 36523847 PMC9745638

[B13] McKhann G. M. Knopman D. S. Chertkow H. Hyman B. T. Jack C. R. Jr. Kawas C. H. (2011). The diagnosis of dementia due to alzheimer's disease: recommendations from the national institute on aging-alzheimer's association workgroups on diagnostic guidelines for alzheimer's disease. Alzheimers Dement. 7, 263–269. 10.1016/j.jalz.2011.03.005 21514250 PMC3312024

[B14] Palmqvist S. Warmenhoven N. Anastasi F. Pilotto A. Janelidze S. Tideman P. (2025). Plasma phospho-tau217 for alzheimer's disease diagnosis in primary and secondary care using a fully automated platform. Nat. Med. 31, 2036–2043. 10.1038/s41591-025-03622-w 40205199 PMC12176611

[B15] Quispialaya K. M. Therriault J. Aliaga A. Benedet A. L. Ashton N. J. Karikari T. (2025). Comparison of plasma p-tau217 and [(18)F]FDG-PET for identifying alzheimer disease in people with early-onset or atypical dementia. Neurology 104, e210211. 10.1212/WNL.0000000000210211 39715476 PMC11666273

[B16] Robinson C. G. Binette A. P. Horie K. Sato C. Schindler S. E. Bateman R. J. (2026). Tau-PET and CSF MTBR-tau243 comparisons validate increased tau aggregation in females. Eur. J. Nucl. Med. Mol. Imaging 53, 5690–5698. 10.1007/s00259-026-07934-y 42191949 PMC13421202

[B17] Rosenberg G. A. (2022). Willis lecture: biomarkers for inflammatory white matter injury in binswanger disease provide pathways to precision medicine. Stroke 53, 3514–3523. 10.1161/STROKEAHA.122.039211 36148658 PMC9613611

[B18] Rosenberg G. A. Cunningham L. A. Wallace J. Alexander S. Estrada E. Y. Grossetete M. (2001). Immunohistochemistry of matrix metalloproteinases in reperfusion injury to rat brain: activation of MMP-9 linked to stromelysin-1 and microglia in cell cultures. Brain Res. 893, 104–112. 10.1016/s0006-8993(00)03294-7 11222998

[B19] Rosenberg G. A. Wallin A. Wardlaw J. M. Markus H. S. Montaner J. Wolfson L. (2016). Consensus statement for diagnosis of subcortical small vessel disease. J. Cereb. Blood Flow. Metab. 36, 6–25. 10.1038/jcbfm.2015.172 26198175 PMC4758552

[B20] Sappey-Marinier D. Calabrese G. Hetherington H. P. Fisher S. N. Deicken R. Van Dyke C. (1992). Proton magnetic resonance spectroscopy of human brain: applications to normal white matter, chronic infarction, and MRI white matter signal hyperintensities. Magnetic Resonance Medicine. 26, 313–327. 10.1002/mrm.1910260211 1513253

[B21] Schneider J. A. Arvanitakis Z. Bang W. Bennett D. A. (2007). Mixed brain pathologies account for Most dementia cases in community-dwelling older persons. Neurology 69, 2197–2204. 10.1212/01.wnl.0000271090.28148.24 17568013

[B22] Snowdon D. A. Greiner L. H. Mortimer J. A. Riley K. P. Greiner P. A. Markesbery W. R. (1997). Brain infarction and the clinical expression of alzheimer disease. The nun study. JAMA. 277, 813–817. 9052711

[B23] Snyder H. M. Corriveau R. A. Craft S. Faber J. E. Greenberg S. M. Knopman D. (2015). Vascular contributions to cognitive impairment and dementia including Alzheimer's disease. Alzheimers Dement. 11, 710–717. 10.1016/j.jalz.2014.10.008 25510382 PMC4731036

[B24] Taheri S. Prestopnik J. Rosenberg G. A. (2024). Barriers of the CNS transfer rate dynamics in patients with vascular cognitive impairment and dementia. Front. Aging Neurosci. 16, 1462302. 10.3389/fnagi.2024.1462302 39385834 PMC11461252

[B25] Toledo J. B. Arnold S. E. Raible K. Brettschneider J. Xie S. X. Grossman M. (2013). Contribution of cerebrovascular disease in autopsy confirmed neurodegenerative disease cases in the national Alzheimer's coordinating centre. Brain 136, 2697–2706. 10.1093/brain/awt188 23842566 PMC3858112

[B26] Wallin A. Sjogren M. Edman A. Blennow K. Regland B. (2000). Symptoms, vascular risk factors and blood-brain barrier function in relation to CT white-matter changes in dementia. Eur. Neurol. 44, 229–235. 10.1159/000008242 11096223

[B27] Wilcock D. Jicha G. Blacker D. Albert M. S. D'Orazio L. M. Elahi F. M. (2021). MarkVCID cerebral small vessel consortium: I. Enrollment, clinical, fluid protocols. Alzheimers Dement. 17, 704–715. 10.1002/alz.12215 33480172 PMC8122220

